# Limited induction of SARS-CoV-2–specific T cell responses in children with multisystem inflammatory syndrome compared with COVID-19

**DOI:** 10.1172/jci.insight.155145

**Published:** 2022-02-22

**Authors:** Vidisha Singh, Veronica Obregon-Perko, Stacey A. Lapp, Anna Marie Horner, Alyssa Brooks, Lisa Macoy, Laila Hussaini, Austin Lu, Theda Gibson, Guido Silvestri, Alba Grifoni, Daniela Weiskopf, Alessandro Sette, Evan J. Anderson, Christina A. Rostad, Ann Chahroudi

**Affiliations:** 1 Department of Pediatrics, Emory University School of Medicine, Atlanta, Georgia, USA.; 2 Center for Childhood Infections and Vaccines of Children’s Healthcare of Atlanta (CHOA) and Emory University, Atlanta, Georgia, USA.; 3 Department of Pathology and Laboratory Medicine, Emory University School of Medicine, Atlanta, Georgia, USA.; 4 Center for Infectious Disease and Vaccine Research, La Jolla Institute for Immunology, La Jolla, California, USA.; 5 Department of Medicine, Emory University School of Medicine, Atlanta, Georgia, USA.

**Keywords:** COVID-19, Adaptive immunity, Cellular immune response, Peptides

## Abstract

Why multisystem inflammatory syndrome in children (MIS-C) develops after SARS-CoV-2 infection in a subset of children is unknown. We hypothesized that aberrant virus–specific T cell responses contribute to MIS-C pathogenesis. We quantified SARS-CoV-2–reactive T cells, serologic responses against major viral proteins, and cytokine responses from plasma and peripheral blood mononuclear cells in children with convalescent COVID-19, in children with acute MIS-C, and in healthy controls. Children with MIS-C had significantly lower virus-specific CD4^+^ and CD8^+^ T cell responses to major SARS-CoV-2 antigens compared with children convalescing from COVID-19. Furthermore, T cell responses in participants with MIS-C were similar to or lower than those in healthy controls. Serologic responses against spike receptor binding domain (RBD), full-length spike, and nucleocapsid were similar among convalescent COVID-19 and MIS-C, suggesting functional B cell responses. Cytokine profiling demonstrated predominant Th1 polarization of CD4^+^ T cells from children with convalescent COVID-19 and MIS-C, although cytokine production was reduced in MIS-C. Our findings support a role for constrained induction of anti–SARS-CoV-2–specific T cells in the pathogenesis of MIS-C.

## Graphical abstract

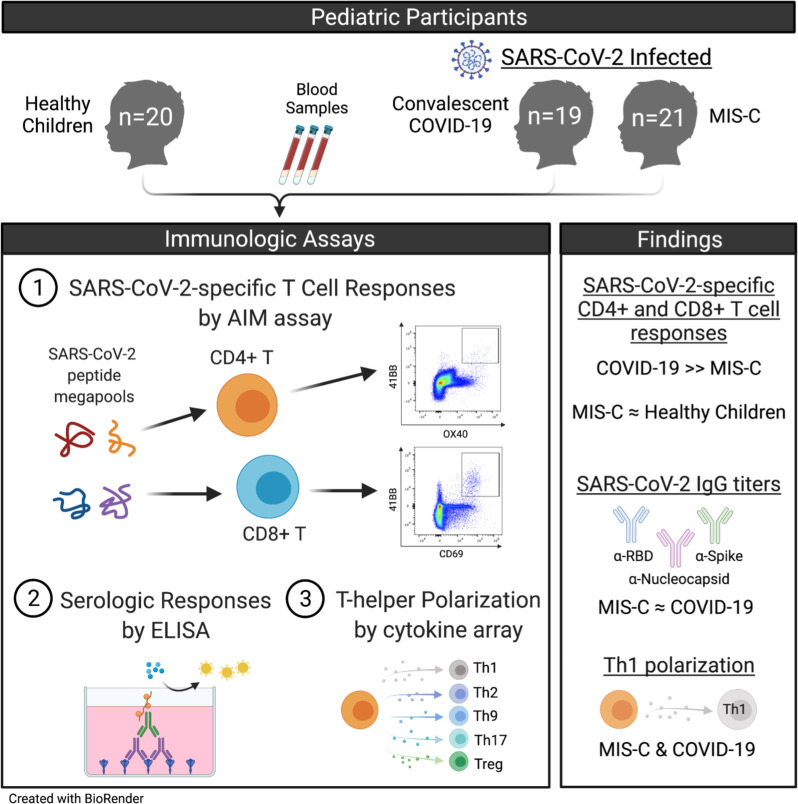

## Introduction

Multisystem inflammatory syndrome in children (MIS-C) is a rare and severe complication of severe acute respiratory syndrome coronavirus 2 (SARS-CoV-2) infection. MIS-C is characterized by serious illness leading to hospitalization, fever, elevated markers of inflammation, multisystem organ involvement, and evidence of infection with SARS-CoV-2 based on reverse transcription PCR (RT-PCR), serology, or antigen test or epidemiologic exposure to persons with COVID-19 (1). Despite the severe presentation of MIS-C, most children have a preceding asymptomatic or mild SARS-CoV-2 infection that often goes unrecognized. Surveillance data in the U.S. from May 2020 through November 2021 shows over 5900 cases of MIS-C reported to the Centers for Disease Control and Prevention (CDC) with disease burden disproportionately affecting Hispanic/Latino, and Black children (2, 3).

The specific mechanisms leading to MIS-C following SARS-CoV-2 infection are unknown, and it can be difficult to distinguish causative factors from those occurring as a consequence of the hyperinflammatory state. However, given the 2- to 8-week delay in MIS-C onset after initial SARS-CoV-2 infection, a role for the adaptive immune system has been hypothesized. We and others have previously found that binding and neutralizing antibody responses to SARS-CoV-2 are robust in hospitalized children with MIS-C (4, 5), although another report described reduced neutralizing responses in MIS-C compared with COVID-19 (6). Enrichment of autoantibodies targeting lymphocyte signaling markers as well as tissues often impacted in MIS-C has been shown (6, 7). Furthermore, T and B cell lymphopenia is a prominent feature of MIS-C (8), with expansion of a subset of T cells utilizing specific TCR Vβ chains, leading to the hypothesis that the presence of a viral reservoir with ongoing viral replication or a viral superantigen drives both immune system dysfunction and immunopathology. It is likely that multiple factors, acting in concert, influence the development of MIS-C.

Here, we investigated the hypothesis that SARS-CoV-2–specific T cells differ in frequency and/or quality in children with MIS-C compared with those with COVID-19. It has been well established that infection with SARS-CoV-2 in adults can initiate strong virus-specific adaptive responses (9, 10), with as high as 100% of adults who have recovered from COVID-19 having detectable memory CD4^+^ T cell responses and 70% having detectable memory CD8^+^ T cell responses (11). Antigen-specific T cell responses in pediatric COVID-19 and MIS-C have not been well characterized. To address this knowledge gap, we analyzed SARS-CoV-2–specific CD4^+^ and CD8^+^ T cells among pediatric participants with MIS-C, COVID-19 (1–2 months after symptom onset), and healthy children (HC). We report here that children with MIS-C demonstrate significantly reduced SARS-CoV-2 CD4^+^ and CD8^+^ T cell responses compared with children with COVID-19, implicating a poorer antiviral T cell response in the development of MIS-C.

## Results

### Study population demographic and clinical history.

Enrollment of children < 21 years of age from CHOA included those with MIS-C (*n* = 21) and COVID-19 (*n* = 19 with blood samples obtained 27–83 days after symptom onset), hereafter referred to as convalescent COVID-19. We collected convalescent blood samples from the COVID-19 participants to approximate a similar interval from SARS-CoV-2 infection as presumed for the MIS-C cohort. Demographics for these cohorts are shown in Table 1. Median age (interquartile range [IQR]) was 9 years (ages 7–12) for children with MIS-C and 15 years (ages 10–17) for those with COVID-19. There were roughly equal distributions of male sex (48% MIS-C, 53% COVID-19). The majority of participants with MIS-C reported Black race and non-Hispanic ethnicity (17 of 21, or 81% for each). Close to half of children with COVID-19 reported Black race (8 of 19 or 42%; *P* = 0.04 compared with MIS-C) and non-Hispanic ethnicity (11 of 19 or 58%; *P* = 0.17 compared with MIS-C).

Clinical and laboratory data were compared for children with MIS-C and COVID-19 at the time of initial diagnosis and/or hospitalization (Table 1). Clinical outcomes for participants with MIS-C were more severe than for those with COVID-19; cardiac or respiratory insufficiency occurred in 76% of children with MIS-C compared with 32% of children with COVID-19 (*P* = 0.01), and 20 of 21 participants with MIS-C were admitted to the intensive care unit (ICU) versus 7 of 19 children with COVID-19 (*P* < 0.0001). There were no deaths in either group. Children with MIS-C presented with significantly higher levels of inflammatory markers, including ferritin and C-reactive protein (CRP) (*P* < 0.01 and *P* < 0.0001, respectively). Mean absolute lymphocyte count (ALC) and platelet count were lower in those with MIS-C than COVID-19 (*P* < 0.0001 and *P* = 0.03, respectively), and mean absolute neutrophil count (ANC) was higher (*P* = 0.01). Nasopharyngeal (NP) specimens tested positive by SARS-CoV-2 RT-PCR in 5 of 21 of those with MIS-C and 19 of 19 of those with COVID-19. All patients with MIS-C who had SARS-CoV-2 nucleocapsid antibody testing were positive. Banked prepandemic and early-pandemic peripheral blood mononuclear cell (PBMC) samples from HC (*n* = 20) without SARS-CoV-2 exposure served as controls. These participants had a median age of 7 years at time of sample collection (IQR, 5–9), were mostly male (65%), were of Black race (80%), and were of non-Hispanic ethnicity (100%). All were confirmed seronegative for SARS-CoV-2 spike receptor binding domain (RBD) IgG by ELISA.

### SARS-CoV-2–specific CD4^+^ T cell responses.

Antigen-specific CD4^+^ T cell responses were measured using the activation induced marker (AIM) assay following stimulation with SARS-CoV-2 T cell peptides (11, 12). Spike reactivity was measured using a megapool (MP) (CD4_S) composed of 253 overlapping 15 mers by 10 amino acids spanning the entire spike protein. CD4^+^ T cell reactivity against the remainder of the SARS-CoV-2 proteome was measured using CD4_R MP based on predicted HLA class II CD4^+^ T cell epitopes and containing 221 peptides from all other non-spike proteins (i.e., membrane, nucleocapsid, envelope, nonstructural proteins, and ORF3a, ORF7a, ORF6, and ORF8). The advantage of the AIM assay is the detection of rare and heterogenous cell populations due to measurement of TCR-dependent upregulation of surface markers compared with cytokine production–dependent techniques that can be less sensitive (13). PBMCs obtained from 21 participants with MIS-C, 19 participants with convalescent COVID-19, and 20 HC were stimulated with CD4^+^ T cell MPs, equimolar volume of DMSO as a negative control, and phytohemagglutinin (PHA) as a positive control (gating strategy shown in Supplemental Figure 1A; supplemental material available online with this article; https://doi.org/10.1172/jci.insight.155145DS1). Baseline levels of activation in the DMSO control condition were similar in COVID-19 and MIS-C groups (Supplemental Figure 1B). In addition, PHA responses were robust and did not differ across groups (Supplemental Figure 1C).

**Figure 1:**
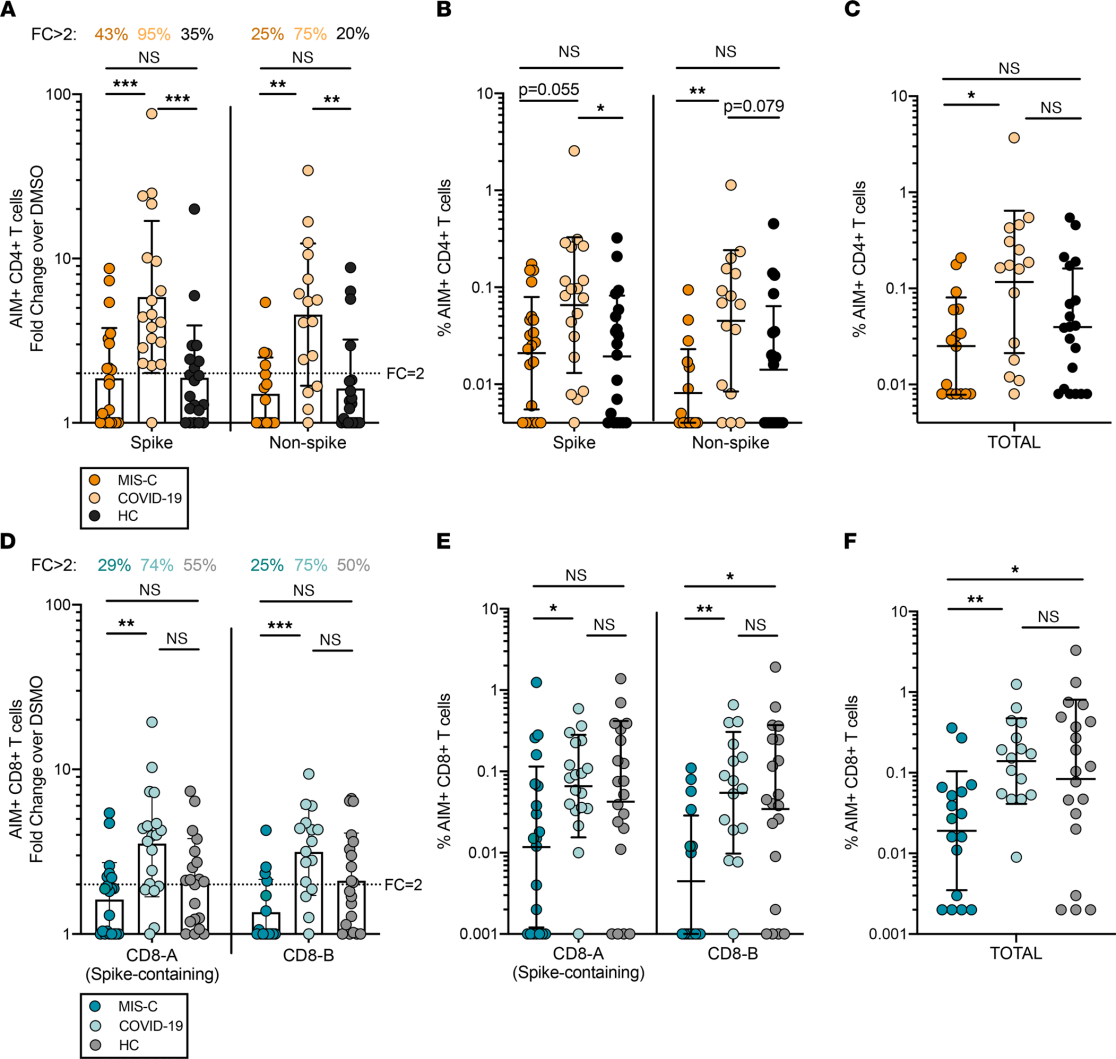
SARS-CoV-2–specific T cell responses. (**A**–**C**) SARS-CoV-2 CD4^+^ T cell data in MIS-C, convalescent COVID-19, and healthy children (HC) shown as AIM^+^ (OX40^+^41BB^+^) CD4^+^ T cells in response to CD4_S peptide MP (spike-containing) and CD4_R peptide MP (non-spike proteins) stimulation by (**A**) fold change (FC) over the DMSO condition, (**B**) frequency of CD4^+^OX40^+^41BB^+^ T cells after MP stimulation with DMSO subtraction, and (**C**) total (frequency of CD4_S plus CD4_R MP) CD4^+^ T cell responses with DMSO subtraction. (**D**–**F**) SARS-CoV-2 CD8^+^ T cell data in MIS-C, convalescent COVID-19, and HC shown as AIM^+^ (CD69^+^41BB^+^) CD8^+^ T cells in response to CD8-A peptide MP and CD8-B peptide MP stimulation by (**D**) FC over the DMSO condition, (**E**) frequency of CD8^+^CD69^+^41BB^+^ T cells after MP stimulation with DMSO subtraction, and (**F**) total (frequency of CD8-A plus CD8-B MP) CD8^+^ T cell responses with DMSO subtraction. DMSO, CD4_S, and CD8-A: MIS-C (*n* = 21), COVID-19 (*n* = 19), and HC (*n* = 20). CD4_R and CD8-B: MIS-C (*n* = 16), COVID-19 (*n* = 16), and HC (*n* = 20). Background-subtracted values ≤ 0 are represented as the lowest calculated AIM^+^ CD4^+^ or CD8^+^ T cell frequency in the data set. Geometric mean shown with statistical comparison by Kruskal-Wallis test. *P < 0.05, **P ≤ 0.01, ***P ≤ 0.001.

CD4^+^ T cell responses (CD4^+^OX40^+^41BB^+^) against SARS-CoV-2 spike and non-spike MPs were detectable in the majority of children with convalescent COVID-19 (95% and 75% with fold change [FC] over DMSO control > 2, respectively; Figure 1A). Children with MIS-C, however, displayed reduced CD4^+^ T cell responses, with 43% exhibiting FC > 2 to SARS-CoV-2 spike MP and only 25% to the non-spike MP (Figure 1A; *P* = 0.0004 and *P* = 0.003 compared with convalescent COVID-19, respectively). The frequency of CD4^+^OX40^+^41BB^+^ T cells trended lower in participants with MIS-C compared with convalescent COVID-19 following spike MP stimulation (*P* = 0.055) and was significantly reduced after non-spike MP stimulation (*P* = 0.01) (Figure 1B), with a lower total CD4^+^OX40^+^41BB^+^ T cell frequency (sum of spike and non-spike responses) in MIS-C versus convalescent COVID-19 (*P* = 0.02) (Figure 1C). Interestingly, no difference in SARS-CoV-2–specific CD4^+^ T cell frequency or FC was observed between children with MIS-C and HC, while convalescent COVID-19 participants had overall higher CD4^+^ T cell responses compared with HC (Figure 1, A–C).

### SARS-CoV-2–specific CD8^+^ T cell responses.

The AIM assay was also utilized to assess antiviral CD8^+^ T cell responses following CD8 MP stimulation. CD8 MPs were derived from epitope prediction for the most frequent groups of HLA class I alleles: CD8-A and CD8-B together include 628 CD8^+^ T cell epitopes, with CD8-A MP containing all structural and some nonstructural protein targets and CD8-B MP containing mostly nonstructural targets (11, 12). Levels of CD8^+^ T cell activation in the DMSO control and following PHA stimulation conditions were similar in COVID-19 and MIS-C groups (Supplemental Figure 1, B and C). As seen with antiviral CD4^+^ T cells, CD8^+^ T cell responses (CD8^+^CD69^+^41BB^+^) against SARS-CoV-2 spike–containing CD8-A MP and CD8-B MP were detectable in the majority of children with convalescent COVID-19 (74% and 75% with FC > 2, respectively; Figure 1D). Children with MIS-C again showed a blunted response, with 29% exhibiting FC > 2 to SARS-CoV-2 CD8-A MP and only 25% to CD8-B MP (Figure 1D; *P* = 0.002 and *P* = 0.0009 compared with convalescent COVID-19, respectively). In contrast, half of HC had detectable CD8^+^ T cell responses (Figure 1D). Those with MIS-C also exhibited a reduced frequency of CD8^+^ T cells responding to CD8-A MP compared with those with convalescent COVID-19 (*P* = 0.045) and had the lowest frequency of CD8^+^ T cells responding to the CD8-B MP epitopes (*P* = 0.007 and *P* = 0.02 compared with convalescent COVID-19 and HC, respectively) (Figure 1E). The total frequency of CD8^+^CD69^+^41BB^+^ T cells (sum of CD8-A and CD8-B MP responses) was lower in participants with MIS-C compared with both convalescent COVID-19 (*P* = 0.008) and HC (*P* = 0.04) (Figure 1F). Spike-specific CD8^+^ T cell responses were also analyzed using the CD4_S MP, and relationships between groups were preserved (not shown).

### Factors influencing variability in SARS-CoV-2–specific T cell responses.

SARS-CoV-2–specific CD4^+^ and CD8^+^ T cell levels were variable, especially in convalescent COVID-19, and we next sought to better understand this range of responses. Although not statistically different, convalescent COVID-19 participants tended to be older than those with MIS-C, so we assessed whether age might contribute to the variable level of antiviral T cells. We found, however, that age did not correlate with CD4^+^ or CD8^+^ T cell responses in either cohort (Supplemental Figure 2). In fact, the convalescent COVID-19 group contained several infants less than 1 year of age, and these all had detectable responses, arguing against younger age in the MIS-C cohort being responsible for the lower levels of antiviral T cells. Additionally, we stratified T cell responses within groups by sex and race, but we did not find these to be influential (Supplemental Figures 3 and 4).

Another factor we considered was disease severity, as severe COVID-19 has been associated with a less robust cellular immune response in adults (14). We, thus, analyzed the T cell responses in convalescent COVID-19 but did not find clear differences between children with mild, moderate, or severe COVID-19 (Supplemental Figure 5A), although numbers were small for each subgroup (Table 2). We next restricted the MIS-C comparison to those with mild COVID-19 based on the premise that, while the majority of children in this study were severely ill when they were hospitalized with MIS-C, all experienced mild or asymptomatic disease in their preceding SARS-CoV-2 infection. Antiviral CD4^+^ and CD8^+^ T cell responses in children convalescing from mild or asymptomatic COVID-19 remained higher than children with MIS-C, with the exception of frequency of spike-specific CD4^+^ T cells (Supplemental Figure 5B).

There were 2 parameters we identified that were associated with SARS-CoV-2–specific T cell responses. First, at baseline, convalescent COVID-19 donors had significantly higher proportions of central and effector memory CD8^+^ T cells and central memory CD4^+^ T cells compared with the MIS-C group (Supplemental Figure 6A), and this may contribute to the weaker antigen-specific T cell activation in MIS-C. In both HC and patients convalescing from COVID-19, but not donors with MIS-C, we observed a positive correlation between CD4^+^ T cell FC to SARS-CoV-2 MPs and the frequency of central memory CD4^+^ T cells (Supplemental Figure 6B). This relationship was preserved for effector memory CD4^+^ T cells in convalescent COVID-19. Similar associations were not found for CD8^+^ T cells. Second, 4 of the 5 (80%) MIS-C patients with positive NP SARS-CoV-2 RT-PCR testing had CD8^+^ T cell responses against both CD8-A and CD8-B MPs with FC < 2, compared with 57% with FC < 2 in the whole MIS-C cohort, irrespective of PCR result. We note that patients with positive RT-PCR results were not sampled closer to symptom onset than the remainder of the group, implicating a relationship between nucleic acid positivity and the weakest CD8^+^ T cell responses in MIS-C.

### Serologic responses in convalescent COVID-19 and MIS-C.

**Figure 2:**
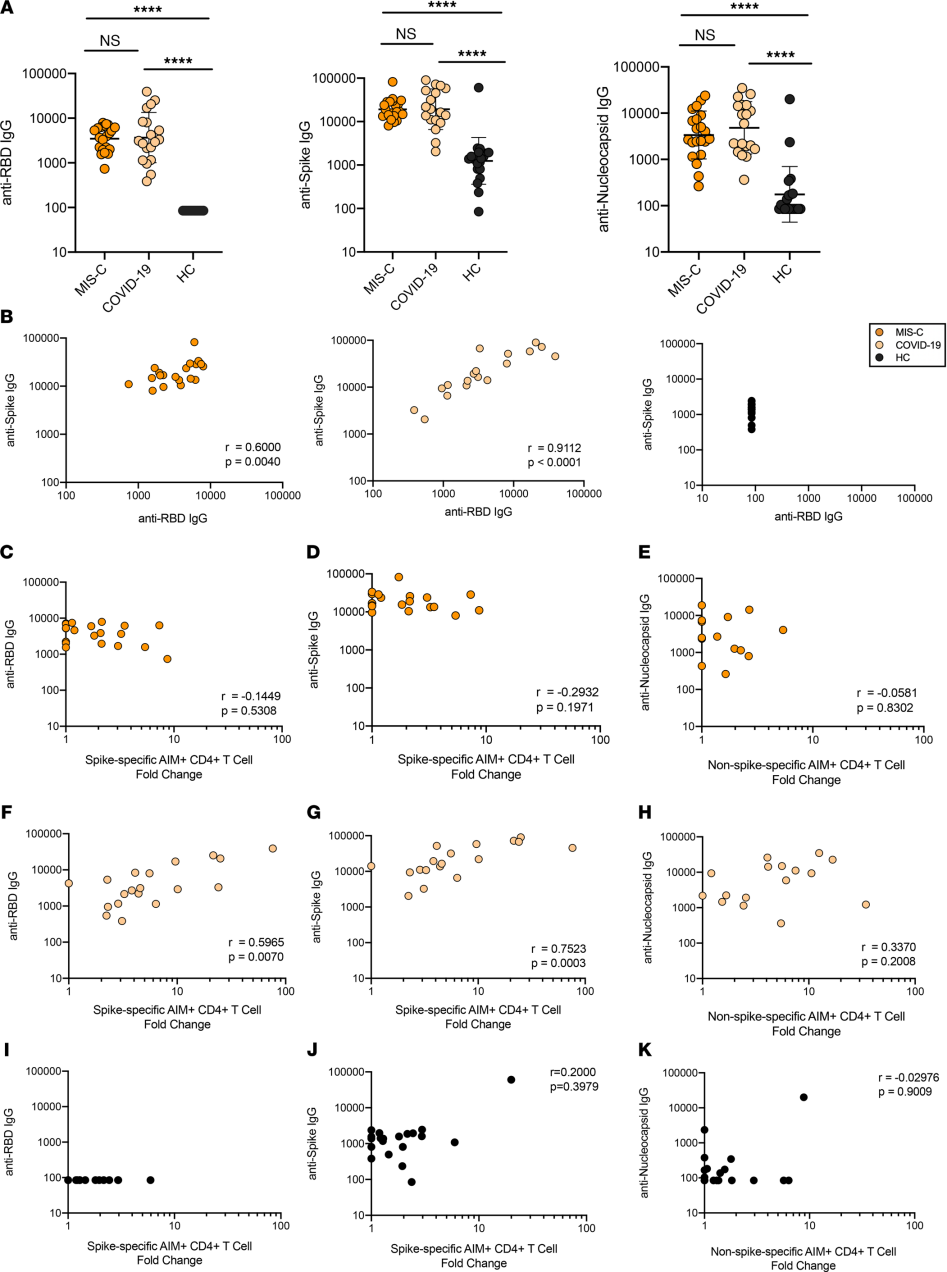
Serologic responses to SARS-CoV-2 antigens and correlations with T cell responses. (**A**) Antibody titers to SARS-CoV-2 RBD, full-length spike, and nucleocapsid protein in MIS-C, convalescent COVID-19, and healthy children (HC). Geometric mean shown with statistical comparison by Kruskal-Wallis test. ****P < 0.0001. (**B**) Relationship between RBD IgG and spike IgG titers for MIS-C, convalescent COVID-19, and HC. (**C**, **D**, **F**, **G**, **I**, and **J**) Relationships between spike-specific CD4^+^ T cell responses (fold change, FC) and RBD IgG titers (**C** and **F**) and full-length spike IgG titers (**D** and **G**) in MIS-C (**C** and **D**), convalescent COVID-19 (**F** and **G**), and HC (**I** and **J**). (**E**, **H**, and **K**) Relationship between non-spike-specific CD4^+^ T cell responses (FC) and nucleocapsid IgG titers for MIS-C (**E**), convalescent COVID-19 (**H**), and HC (**K**). Statistical relationships in **C**–**K** were assessed with Spearman’s correlation.

We previously reported that antibody responses to SARS-CoV-2 in children hospitalized with MIS-C were higher than those in children with acute COVID-19, and as found in adults (14, 15), neutralizing titers strongly correlated with RBD IgG levels in children (4). Here, we compared serologic responses to SARS-CoV-2 RBD, full-length spike, and nucleocapsid in children with convalescent COVID-19, MIS-C, and HC and assessed for correlations with CD4^+^ T cell responses. MIS-C and convalescent COVID-19 participants had similar geometric mean titers against all tested viral antigens that were significantly higher than those of HC (*P* < 0.0001 for each comparison) (Figure 2A). Similar to our cellular immune response results, an association between antibody titer and COVID-19 disease severity in this cohort of children was not observed (Supplemental Figure 7). As expected, we found that RBD and spike titers correlated with each other in children with MIS-C (*r* = 0.6; *P* = 0.004) and convalescent COVID-19 (*r* = 0.91; *P* < 0.0001) — but not in HC (Figure 2B). CD4^+^ T cell responses to the spike MP correlated with both RBD IgG titers (*r* = 0.6; *P* = 0.007) and spike IgG titers (*r* = 0.75; *P* = 0.0003) in participants with convalescent COVID-19 (Figure 2, F and G) but not MIS-C (Figure 2, C and D). Nucleocapsid antibody levels did not correlate with CD4^+^ T cell responses to the non-spike MP in either group (Figure 2, E and H), perhaps because this MP contains multiple other antigenic targets in addition to nucleocapsid. Similar to the MIS-C group, CD4^+^ T cell responses did not correlate with antibody titers in HC (Figure 2, I–K).

### CD4^+^ T cell cytokine profiling.

**Figure 3:**
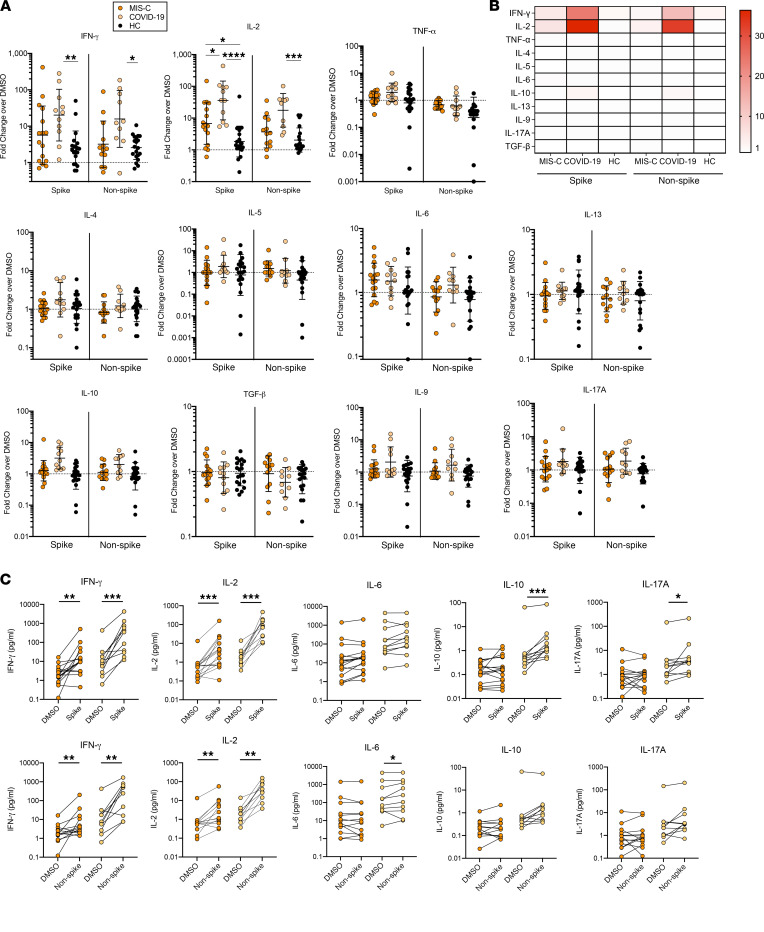
SARS-CoV-2–specific CD4^+^ T cell cytokine profiling. (**A**) Cytokine production for major T helper cytokines shown as fold change (FC) of cytokine concentration after CD4_ S MP (spike-containing) and CD4_R MP (non-spike) stimulation versus DMSO control measured on a subset of participants by MSD multiplex cytokine array. Individual results for MIS-C (*n* = 19), convalescent COVID-19 (*n* = 12), and HC (*n* = 20) are plotted with geometric mean ± SDs. Statistical comparison between groups was conducted by Kruskal-Wallis test. (**B**) Heatmap showing CD4^+^ T cell cytokine response by median FC in cytokine production for each peptide MP by clinical cohort. (**C**) Cytokine concentrations from DMSO control and pair-matched peptide stimulation (Top, spike-containing MP; bottom, non-spike MP) for MIS-C and convalescent COVID-19. Cytokines with significant increases after stimulation are shown. Statistical comparison using Wilcoxon matched-pairs signed rank test. *P < 0.05, **P < 0.01, ***P < 0.001, ****P < 0.0001.

Finally, to evaluate CD4^+^ T helper polarization, we performed cytokine arrays on cell culture supernatants from the CD4^+^ T cell MP stimulations in the AIM assay. We found that children with convalescent COVID-19 produced cytokines typical of a Th1 response (IFN-γ and IL-2) following stimulation with both spike and non-spike antigens (Figure 3A). The same was true of children with MIS-C, although as with activation marker expression, reduced levels of these cytokines were found in MIS-C compared with convalescent COVID-19 (Figure 3, A and B). Cytokines typical of Th2, Th9, Th17, and Tregs were either low or undetectable, although IL-6, IL-10, and IL-17A levels were higher in peptide-stimulated conditions compared with DMSO control conditions for convalescent COVID-19 participants (Figure 3C). These results are consistent with a blunted CD4^+^ T cell response, rather than skewed Th cell polarization, in children with MIS-C.

## Discussion

MIS-C is a severe outcome of SARS-CoV-2 infection in children, and while still considered rare, over 5,900 children have been diagnosed with MIS-C in the United States. Accordingly, there has been a heightened effort to discover the pathogenesis of MIS-C to better understand who may be at risk and to develop targeted therapeutics. Here, we investigated the simple hypothesis that MIS-C may develop in part due to an ineffective cellular immune response against SARS-CoV-2. We show that both CD4^+^ and CD8^+^ antigen-specific T cell responses were reduced in children with MIS-C compared with those convalescing from COVID-19 and were similar to or lower than responses in HC. These results fit with a model whereby an ineffective antiviral response after SARS-CoV-2 infection may result in reduced systemic viral clearance, persistent antigen expression, and resultant hyperinflammation in children who go on to develop MIS-C.

SARS-CoV-2 specific CD4^+^ and CD8^+^ T cells have been measured in multiple cohorts of adults with COVID-19 (9–12, 16). Here, we studied pediatric participants in the convalescent phase of COVID-19 (1–2 months from symptom onset) to match the approximate time from SARS-CoV-2 infection to MIS-C development. Studies in adult populations that have included convalescent time points demonstrate that about 70%–90% of adults with COVID-19 have detectable CD4^+^ and CD8^+^ T cell responses 1 month after symptom onset (17), which is remarkably similar to what we found in children. Higher levels of antigen-specific T cell responses are associated with reduced COVID-19 disease severity in adults (14), and antiviral T cells generated through vaccination are presumed to contribute to the reductions in COVID-19–related hospitalizations and deaths seen in multiple clinical trials (9, 18–21). We did not observe higher T cell responses in pediatric participants with mild COVID-19 as compared with moderate or severe COVID-19. However, the reduced T cell response in MIS-C compared with COVID-19 at a similar time after infection suggests that diminished circulating SARS-CoV-2–specific T cell responses may promote MIS-C immunopathogenesis.

A recent investigation into potential drivers of MIS-C reported persistence of SARS-CoV-2 RNA in the stool, associated intestinal hyperpermeability, and spike and S1 protein antigenemia that did not decline with seroconversion (22). Indeed, patients with MIS-C have a predominance of GI symptoms. Yonker et al. (22) theorize that the hyperinflammatory state of MIS-C develops in individuals in whom viral replication in the GI tract continues long after initial infection, followed by breaches in the intestinal barrier and release of superantigen-containing spike protein (23) into the systemic circulation that triggers inflammatory cytokine production. The weak antiviral T cell responses we report in children with MIS-C could be a factor leading to unchecked SARS-CoV-2 replication in the gut. Although we did not measure virus in the stool here, we did observe an increased proportion of respiratory PCR positivity in MIS-C patients with the lowest CD8^+^ T cell levels. Prolonged antigen exposure may further result in exhaustion and apoptosis of SARS-CoV-2–specific T cells in MIS-C, and indeed, patients with MIS-C have increased PD-1 expression on CD4^+^ and CD8^+^ T cells, along with the hallmark feature of low ALC.

The humoral immune response is intact in patients with MIS-C, as evidenced by high levels of IgG antibodies to SARS-CoV-2 RBD, spike, and nucleocapsid shown here and elsewhere (4, 5, 7, 24). We previously showed that RBD IgG and neutralizing antibodies are highly correlated in children (4). In addition, children hospitalized with acute COVID-19 had significantly lower levels of these antibodies compared with those with MIS-C (4); however, the convalescent COVID-19 and MIS-C participants included in the current study had similar titers, supporting the expected timeline of seroconversion after infection. The intact antibody response argues for preservation of CD4^+^ follicular T cell help for B cells in lymph nodes, although we did not measure this directly. Interestingly, while circulating CD4^+^ T cells against spike epitopes directly correlated with RBD- and spike-specific IgG in pediatric COVID-19, this was not the case for MIS-C. Reasons may include the lower levels of antiviral T cells, the mild or asymptomatic initial infection that universally characterized our MIS-C cohort, or a combination of these (or other) factors.

Several studies have provided evidence for additional pathogenic mechanisms involved in MIS-C. As with most investigations of MIS-C, these are complicated by the fact the initial SARS-CoV-2 infection is typically asymptomatic or mild, meaning that patients are not usually identified for research purposes until well into the pathogenic course. It can, therefore, be difficult to separate the cause versus consequence of hyperinflammation seen in MIS-C. Clinical markers of inflammation (CRP, erythrocyte sedimentation rate [ESR], ferritin) that define MIS-C are accompanied by inflammatory cytokines and innate immune activation signals (25–27). Due to the delayed onset of MIS-C symptoms, it has been proposed that autoantibodies that develop following SARS-CoV-2 infection may play a role in disease (6, 7, 28). Other work has implicated a dysregulated immune response, with elevated plasmablast frequencies and increased activation of certain subsets of CD8^+^ T cells in MIS-C compared with pediatric COVID-19 (8). We did not observe increased CD4^+^ or CD8^+^ T cell activation at baseline in MIS-C compared with COVID-19 (Supplemental Figure 1B); a finding that may be explained by differences in study populations and/or experimental conditions between our work at that of Vella et al. (8). An immune profiling study of patients with MIS-C also revealed plasmablast expansion, increased cytotoxicity genes in CD8^+^ T and NK cells, endothelial reactive antibodies, and a skewed TCR repertoire in memory T cells (26). Enrichment of TRBV11-2 in memory T cells (CD4^+^ and CD8^+^) in severe MIS-C may reflect autoreactive T cells or response to a superantigen (26, 29). Our data suggest that TCR expansions found in MIS-C are not due to an enhanced cellular immune response directed against SARS-CoV-2.

Two prior studies have evaluated T cell responses in children with MIS-C using intracellular cytokine staining for IFN-γ and compared them with patients with COVID-19 (30, 31). Moreews et al. showed a poor response to SARS-CoV-2 peptide stimulation in MIS-C compared with adults with convalescent COVID-19 (30). Pierce et al. found similar levels of spike-specific CD4^+^ T cells in pediatric MIS-C and COVID-19, and both groups did not differ from healthy controls (31). This result contrasts with our findings, possibly related to the use of acute COVID-19 samples, as well as the different stimulations and functional read-outs. The advantage of the MPs we used is that T cell responses directed against the full complement of viral structural and nonstructural proteins can be assessed. Recently, Hsieh et al. employed the same MPs to assess T cell responses in 11 patients with MIS-C and found detectable responses (FC > 2) in a higher proportion than we observed here, although frequencies of responding T cells were similar (32). A comparable group of pediatric COVID-19 patients was not included, thus limiting our ability to contextualize their findings with our results. A strength of our study is the inclusion of appropriate pediatric control groups permitting the relative comparisons across cohorts that revealed the reduced SARS-CoV-2–specific T cells in MIS-C versus convalescent COVID-19.

Interestingly, half of HC had CD8^+^ T cells specific for SARS-CoV-2 antigens, particularly notable for the SARS-CoV-2 nonstructural epitopes where a higher frequency of CD8^+^ T cells was seen compared with MIS-C. A lower proportion (20%–35%) of HC had SARS-CoV-2–reactive CD4^+^ T cells. HC had similar positive control PHA responses as those with COVID-19 and MIS-C (Supplemental Figure 1C) and had low levels of activated T cells at baseline (Supplemental Figure 1B). HC did have a higher frequency of memory CD8^+^ T cells and lower frequency of naive CD8^+^ T cells compared with the MIS-C, but not COVID-19 group (Supplemental Figure 6). We confirmed that all HC were seronegative for RBD IgG, indicating lack of exposure to SARS-CoV-2. Presumably, the T cell responses in HC reflect cross-reactivity with common cold coronaviruses. This cross-reactivity may also explain the few HC who displayed high spike and nucleocapsid IgG titers. Both humoral and cellular immune responses that are cross-reactive with the common cold coronaviruses have been described, mostly within CD4^+^ T cells (33). Children have been previously infected with fewer coronaviruses than adults based upon years of life; however, endemic coronavirus infection in children may have been more recent, potentially accounting for the high T cell responses seen here in HC. Preexisting SARS-CoV-2–specific T cell responses have also been linked to certain HLA haplotypes (34). Although there is some evidence to the contrary (35), at least in adults, preexisting, cross-reactive antibodies do not appear to protect against SARS-CoV-2 infection (36). In contrast, a recent study of healthcare workers implicates coronavirus polymerase-specific T cells in abortive SARS-CoV-2 infection (37).

Limitations of this work include the small number of participants, which may have affected our ability to detect differences when evaluating subgroups (by demographic factors, for instance). In addition, while studying participants 1–2 months after COVID-19 symptoms approximated the expected time frame between SARS-CoV-2 infection and onset of MIS-C, we were not able to precisely date infection in the MIS-C group, potentially leading us to sample the COVID-19 and MIS-C cohorts at slightly different times since infection. Furthermore, most blood samples from patients with MIS-C were collected after receipt of treatments, including IVIg and/or corticosteroids, although we did not observe any statistically significant differences in T cell responses between those who received steroids prior to enrollment and those who did not. We were unable to finely epitope map the T cell responses and phenotypically and functionally characterize antigen-specific T cells in greater detail, a limitation related to restrictions on pediatric blood volume collection, especially in children hospitalized with MIS-C who receive very frequent laboratory assessments. Recognizing that invasive sampling in children is challenging, we note that analysis of peripheral blood does not provide insight into tissue-localized immune responses, nor homing of T cells from circulation to mucosal sites (38). Longitudinal studies where blood is collected in the convalescent phase of MIS-C, as well as COVID-19, are underway that should permit these more refined analyses, including assessment of trafficking receptor expression on T cells.

In summary, while it is likely that multiple pathogenic mechanisms contribute to the immune activation and inflammation that characterizes MIS-C, our data reveal constrained induction of anti–SARS-CoV-2–specific CD4^+^ and CD8^+^ T cells as an instigator of this rare but life-threatening outcome of SARS-CoV-2 infection in children. If MIS-C is indeed driven by a weak and/or dysregulated adaptive T cell immune response responsible for ineffective viral clearance leading to persistent antigen expression, then targeted therapies to reduce viral burden and/or boost T cell responses (e.g., vaccines) may be effective in preventing MIS-C.

## Methods

### Study populations.

With approval from the Emory University and CHOA IRBs, blood from children (0–20 years of age) with MIS-C or convalescing from COVID-19 was obtained from April 2020 to February 2021 following informed consent and assent, as appropriate for age. Detailed overview of patient characteristics is shown in Table 1. The CDC case definition was used to identify MIS-C (39). For children with COVID-19, blood was collected in the convalescent phase of infection (27–83 days from illness onset), and disease severity was determined based on criteria shown in Table 2. Blood from MIS-C patients collected during hospitalization, with the exception of a single MIS-C donor whose blood was sampled at 53 days after MIS-C symptom onset due to inadvertent misclassification as COVID-19. This individual was retained in the study as days from MIS-C symptom onset was not consistently associated with the level of SARS-CoV-2–specific T cell responses (Supplemental Figure 8). The majority of MIS-C samples was collected after IVIg (90%) and/or corticosteroid (67%) treatment due to the time required for obtaining informed consent and patients requiring immediate medical care. Antiviral T cell responses were not significantly different in patients with MIS-C who did or did not receive corticosteroid treatment (Supplemental Figure 9). Banked samples from HC collected before the pandemic or early in the pandemic, with undetectable SARS-CoV-2 RBD antibodies, were included as controls.

### AIM assay.

Quantification of SARS-CoV-2–specific T cell responses was done by AIM assay, as previously described (11). In brief, cryopreserved PBMC were thawed and rested overnight in an inclined conical tube at 4 million cells/mL. Rested cells were resuspended in RPMI-5% AB serum and added to a 96-well round-bottom plate, placing approximately 1 million cells per well. Cells were stimulated at 1 μg/mL with 4 different SARS-CoV-2–specific peptide MP provided by Alba Grifoni at the La Jolla Institute in Ja Jolla, California, USA: CD4_S MP (spike), CD4_R MP (non-spike), CD8-A (spike containing), or CD8-B (non-spike containing). PHA-P (10 μg/mL) (Millipore Sigma) and equimolar DMSO conditions were included as positive and negative controls, respectively. After 24 hours, cells were washed and stained in the plate using: Live/Dead Fixable Aqua (Thermo Fisher Scientific), CD14-AF700 (M5E2, BioLegend), CD20-AF700 (2H7, Thermo Fisher Scientific), CD3-APC-Cy7 (OKT3, BioLegend), CD4-BV605 (SK3, BioLegend), CD8-BV711 (SK1, BioLegend), CD69-PE-CF594 (FN50, BD Biosciences), 4-1BB-APC (4B4-1, BioLegend), OX40-PE-Cy7 (Ber-ACT35, BD Biosciences), C45RA-BB700 (5H9, BD Biosciences), and CCR7-FITC (150503, BD Biosciences). Incubation with viability dye and CCR7 was at 37°C for 15 minutes; all other markers were incubated at 4°C for 50 minutes. Samples were washed twice with FACS buffer and treated with 2% PFA for 15 minutes at 4°C. After 2 final washes, cells were resuspended in FACS buffer and acquired on a 4-laser Cytek Aurora using SpectroFlo software (Cytek Biosciences). Analysis was performed using FlowJo v10 (BD Biosciences).

### Cytokine bead array.

Cytokine profiling was performed on a subset of 16 MIS-C, 11 COVID-19, and 20 HC. Supernatants from AIM assay cultures were harvested 24 hours after stimulation for cytokine analysis using the U-PLEX platform (Meso Scale Discovery [MSD]). The array was customized for evaluation of CD4^+^ T helper responses, which included multiplex detection of IFN-γ, IL-2, IL-4, IL-5, IL-6, IL-9, IL-10, IL-13, IL-17A, and TNF-α. The assay was performed on undiluted supernatant (25 μL) in duplicate per the manufacturer’s recommendation. Samples were read on a MESO Quickplex SQ 120 (MSD) and quantified using calibration standards of known concentration.

### SARS-CoV-2 serology.

RBD, spike, and nucleocapsid IgG antibodies against SARS-CoV-2 were measured in plasma or serum by ELISA. ELISAs were carried out as described by Suthar et al., with few modifications described here (15). Each coating antigen (recombinant RBD, purified full-length spike, and purified nucleocapsid protein was coated on Nunc MaxiSorp plates at 1 µg/mL in 100 µL of PBS and incubated at 4ºC overnight. Plates were blocked in PBS/0.05% Tween/1% BSA ELISA buffer for 2 hours at room temperature (RT). Patient plasma or serum and controls were kept on ice and first diluted 1:100 in ELISA buffer; they were then serially diluted 1:3. Diluted samples were added 100 μL/well and incubated for 90 minutes at RT. Secondary goat–anti-hu HRP IgG was diluted in ELISA buffer at 1:5000, and 100 μL/well was added for 1 hour at RT. Plates were developed using 0.4 g/L o-phenylenediamine substrate in 0.05M phosphate-citrate buffer with 0.012% hydrogen peroxide, and reactions were quenched with 1M HCl. Plates were washed 4 times with 300 μL of PBS/0.05%Tween between steps. Absorbance was read at 490 nm, absorbance curves were generated by using nonlinear regression analysis, and endpoint titers were interpolated from curves using a baseline value calculated from the pooled sera of 8 HC. The assay LOD was 100, and undetectable titers were assigned a value of 85.

### Statistics.

All graphs and statistical analyses were done on Prism v8 (GraphPad). Differences in patient characteristics and clinical parameters between MIS-C and COVID-19 were evaluated by 2-tailed unpaired *t* test for continuous data and Fisher’s exact test or Pearson’s χ^2^ for categorical data. Kruskal-Wallis test with Dunn’s correction for multiple comparisons was used to compare ELISA data and the frequency and FC of AIM^+^ cells between the 3 patient groups. Mann-Whitney *U* test was used for comparing median frequency and FC of AIM^+^ cells between MIS-C and COVID-19. Correlations between immune parameters were assessed by Spearman correlation. *P* < 0.05 was considered statistically significant. In all graphs, asterisks indicate the following: *P < 0.05, **P < 0.01, ***P < 0.001, ****P < 0.0001.

### Study approval.

Approval from the Emory University and CHOA IRBs and written informed consent were received prior to study participation.

## Author contributions

AC, VOP, and VS conceived and designed the study and wrote the manuscript. VS performed the T cell experiments, analyzed immunologic data, and conducted statistical analyses. CAR, SAL, AMH, and AB conducted serologic assays and analysis of serologic data. Patient enrollment was coordinated and clinical data was acquired by TG, LM, LH, and AL. AG, DW, and AS provided SARS-CoV-2 peptide MP. AC, VOP, CAR, EJA, GS, AB, DW, and AS reviewed and edited the manuscript.

## Supplementary Material

Supplemental data

## Figures and Tables

**Table 1:**
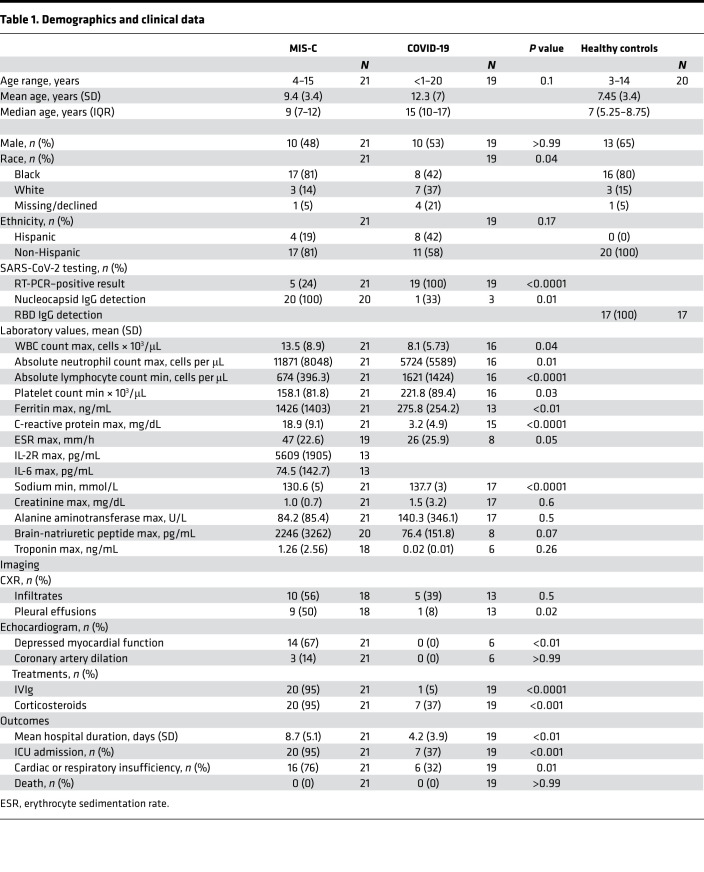
Demographics and clinical data

**Table 2:**
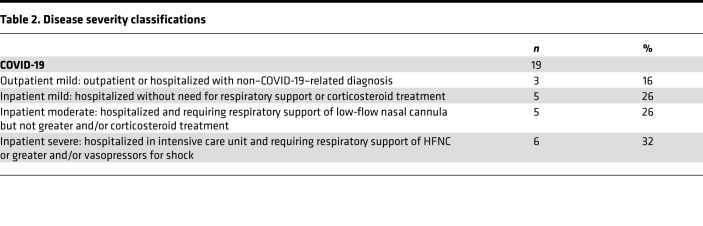
Disease severity classifications

## Version 1 01/19/2022

In-Press Preview

## Version 2 02/22/2022

Electronic publication

## References

[1] Godfred-Cato S (2020). COVID-19-associated multisystem inflammatory syndrome in children — United States, March-July 2020. MMWR Morb Mortal Wkly Rep.

[2] Feldstein LR (2021). Characteristics and outcomes of US children and adolescents with multisystem inflammatory syndrome in children (MIS-C) compared with severe acute COVID-19. JAMA.

[3] https://www.cdc.gov/mis-c/cases/index.html.

[4] Rostad CA (2020). Quantitative SARS-CoV-2 serology in children with multisystem inflammatory syndrome (MIS-C). Pediatrics.

[5] Anderson EM (2021). Severe acute respiratory syndrome-coronavirus-2 (SARS-CoV-2) antibody responses in children with multisystem inflammatory syndrome in children (MIS-C) and mild and severe coronavirus disease (COVID-19). J Pediatric Infect Dis Soc.

[6] Gruber CN (2020). Mapping systemic inflammation and antibody responses in multisystem inflammatory syndrome in children (MIS-C). Cell.

[7] Consiglio CR (2020). The immunology of multisystem inflammatory syndrome in children with COVID-19. Cell.

[8] Vella LA (2021). Deep immune profiling of MIS-C demonstrates marked but transient immune activation compared to adult and pediatric COVID-19. Sci Immunol.

[9] Sekine T (2020). Robust T cell immunity in convalescent individuals with asymptomatic or mild COVID-19. Cell.

[10] Braun J (2020). SARS-CoV-2-reactive T cells in healthy donors and patients with COVID-19. Nature.

[11] Grifoni A (2020). Targets of T cell responses to SARS-CoV-2 coronavirus in humans with COVID-19 disease and unexposed individuals. Cell.

[12] Grifoni A (2020). A sequence homology and bioinformatic approach can predict candidate targets for immune responses to SARS-CoV-2. Cell Host Microbe.

[13] Reiss S (2017). Comparative analysis of activation induced marker (AIM) assays for sensitive identification of antigen-specific CD4 T cells. PLoS One.

[14] Rydyznski Moderbacher C (2020). Antigen-specific adaptive immunity to SARS-CoV-2 in acute COVID-19 and associations with age and disease severity. Cell.

[16] Weiskopf D (2020). Phenotype and kinetics of SARS-CoV-2-specific T cells in COVID-19 patients with acute respiratory distress syndrome. Sci Immunol.

[17] Dan JM (2021). Immunological memory to SARS-CoV-2 assessed for up to 8 months after infection. Science.

[18] Polack FP (2020). Safety and efficacy of the BNT162b2 mRNA Covid-19 vaccine. N Engl J Med.

[19] Baden LR (2021). Efficacy and safety of the mRNA-1273 SARS-CoV-2 vaccine. N Engl J Med.

[20] Thompson MG (2021). Interim estimates of vaccine effectiveness of BNT162b2 and mRNA-1273 COVID-19 vaccines in preventing SARS-CoV-2 infection among health care personnel, first responders, and other essential and frontline workers — eight U.S. locations, December 2020-March 2021. MMWR Morb Mortal Wkly Rep.

[21] Sadoff J (2021). Interim results of a phase 1-2a trial of Ad26.COV2.S covid-19 vaccine. N Engl J Med.

[22] Yonker LM (2021). Multisystem inflammatory syndrome in children is driven by zonulin-dependent loss of gut mucosal barrier. J Clin Invest.

[23] Cheng MH (2020). Superantigenic character of an insert unique to SARS-CoV-2 spike supported by skewed TCR repertoire in patients with hyperinflammation. Proc Natl Acad Sci U S A.

[24] Weisberg SP (2021). Distinct antibody responses to SARS-CoV-2 in children and adults across the COVID-19 clinical spectrum. Nat Immunol.

[25] Carter MJ (2020). Peripheral immunophenotypes in children with multisystem inflammatory syndrome associated with SARS-CoV-2 infection. Nat Med.

[26] Ramaswamy A (2021). Immune dysregulation and autoreactivity correlate with disease severity in SARS-CoV-2-associated multisystem inflammatory syndrome in children. Immunity.

[27] Peart Akindele N (2021). Distinct cytokine and chemokine dysregulation in hospitalized children with acute COVID-19 and multisystem inflammatory syndrome with similar levels of nasopharyngeal SARS-CoV-2 shedding. J Infect Dis.

[28] Porritt RA (2021). The autoimmune signature of hyperinflammatory multisystem inflammatory syndrome in children. J Clin Invest.

[29] Porritt RA (2021). HLA class I-associated expansion of TRBV11-2 T cells in multisystem inflammatory syndrome in children. J Clin Invest.

[30] Moreews M (2021). Polyclonal expansion of TCR Vbeta 21.3^+^ CD4^+^ and CD8^+^ T cells is a hallmark of multisystem inflammatory syndrome in Children. Sci Immunol.

[31] Pierce CA (2020). Immune responses to SARS-CoV-2 infection in hospitalized pediatric and adult patients. Sci Transl Med.

[32] Hsieh LE Characterization of SARS-CoV-2 and common cold coronavirus-specific T-cell responses in MIS-C and Kawasaki disease children. Eur J Immunol.

[33] Niessl J (2021). T cell immunity to SARS-CoV-2. Semin Immunol.

[34] Francis JM Allelic variation in class I HLA determines CD8^+^ T cell repertoire shape and cross-reactive memory responses to SARS-CoV-2. Sci Immunol.

[35] Ng KW (2020). Preexisting and de novo humoral immunity to SARS-CoV-2 in humans. Science.

[36] Anderson EM (2021). Seasonal human coronavirus antibodies are boosted upon SARS-CoV-2 infection but not associated with protection. Cell.

[37] Swadling L (2022). Pre-existing polymerase-specific T cells expand in abortive seronegative SARS-CoV-2. Nature.

[38] Poon MML (2021). SARS-CoV-2 infection generates tissue-localized immunological memory in humans. Sci Immunol.

[39] https://emergency.cdc.gov/HAN/.

