# Supplementary material for: Limited induction of SARS-CoV-2–specific T cell responses in children with multisystem inflammatory syndrome compared with COVID-19
Source: JCI Insight. 2022 Feb 22;7(4):e155145. doi: 10.1172/jci.insight.155145 (PMC8876428; doi:10.1172/jci.insight.155145)
Supplement: Supplemental data [file jciinsight-7-155145-s051.pdf]

Supplemental Information

Figure S1.

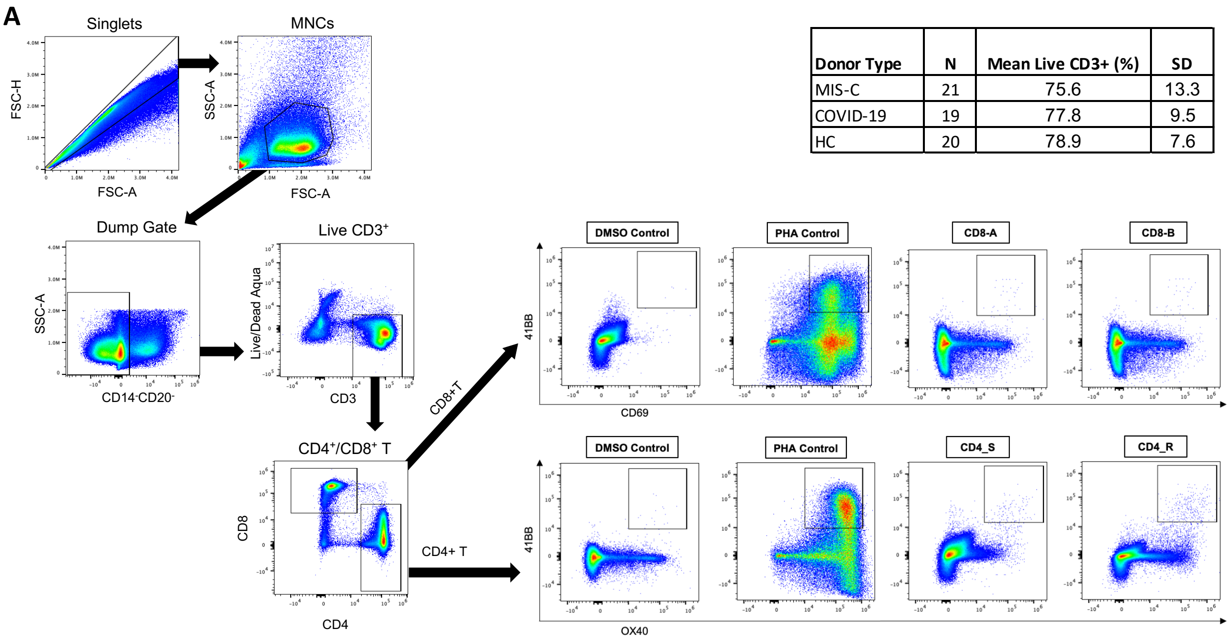

Donor Type	N	Mean Live CD3+ (%)	SD
MIS-C	21	75.6	13.3
COVID-19	19	77.8	9.5
HC	20	78.9	7.6

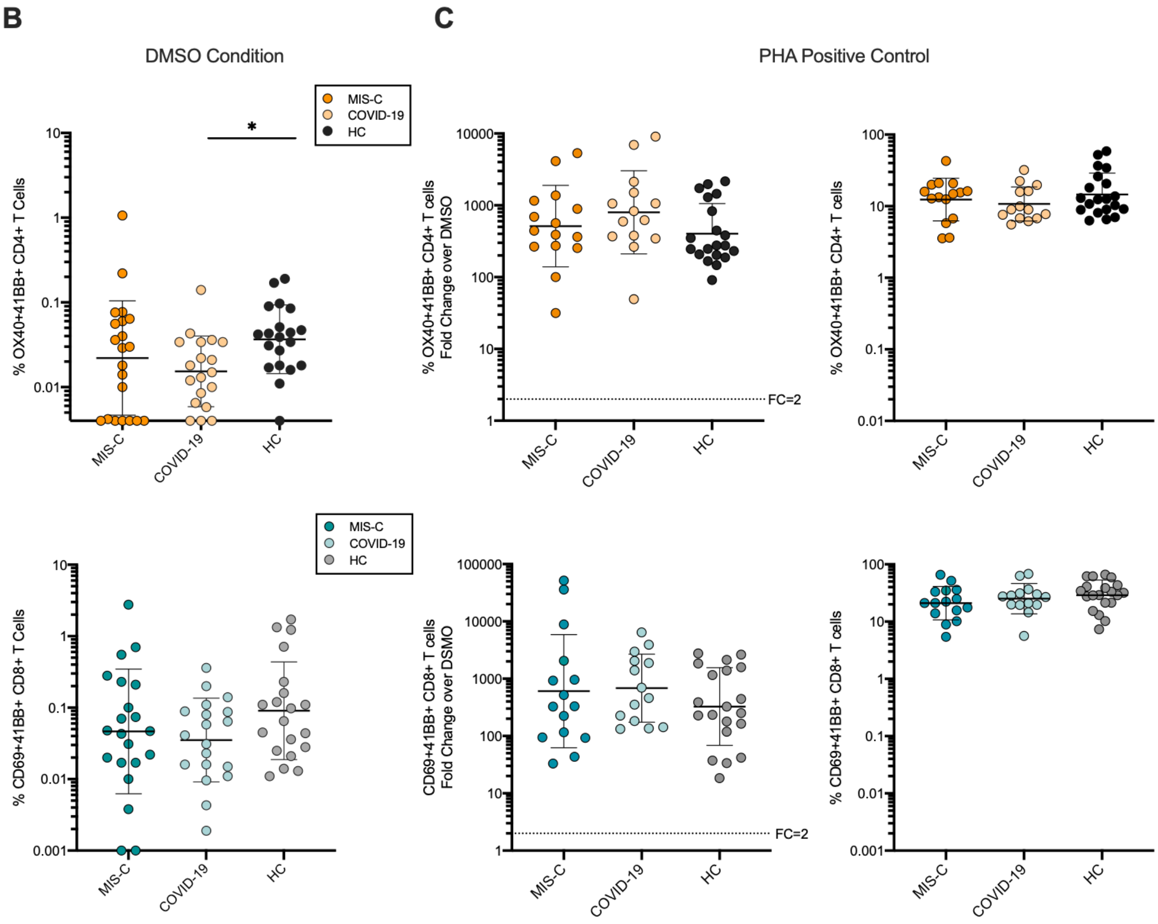

Figure S1. AIM Assay Gating Strategy and Controls. (A) Representative gating strategy to determine frequency of live CD4+ and CD8+ T-cells after DMSO negative control, PHA positive control, and peptide MP stimulation conditions. A convalescent COVID-19 participant is shown. Table shows mean T-cell viabilities in AIM assay across donors (% of live CD3+ T-cells). No significant differences found by Kruskal-Wallis test. (B) T-cell responses to negative control condition DMSO in MIS-C, convalescent COVID-19, and healthy children (HC) shown as frequency of OX40+41BB+CD4+ and CD69+41BB+ CD8+ T-cells; MIS-C (n=21), COVID-19 (n=19), and HC (n=20). (C) T-cell responses to positive control stimulation with PHA shown as OX40+41BB+CD4+ and CD69+41BB+CD8+ T-cell fold change (FC) over DMSO (L plots) and frequency with DMSO subtraction (R plots); MIS-C (n=15), COVID-19 (n=14), and HC (n=20). Geometric mean and geometric mean standard deviation (SD) shown, and only those with statistically significant relationships by Kruskal-Wallis test shown. * $p<0.05$.

Figure S2.

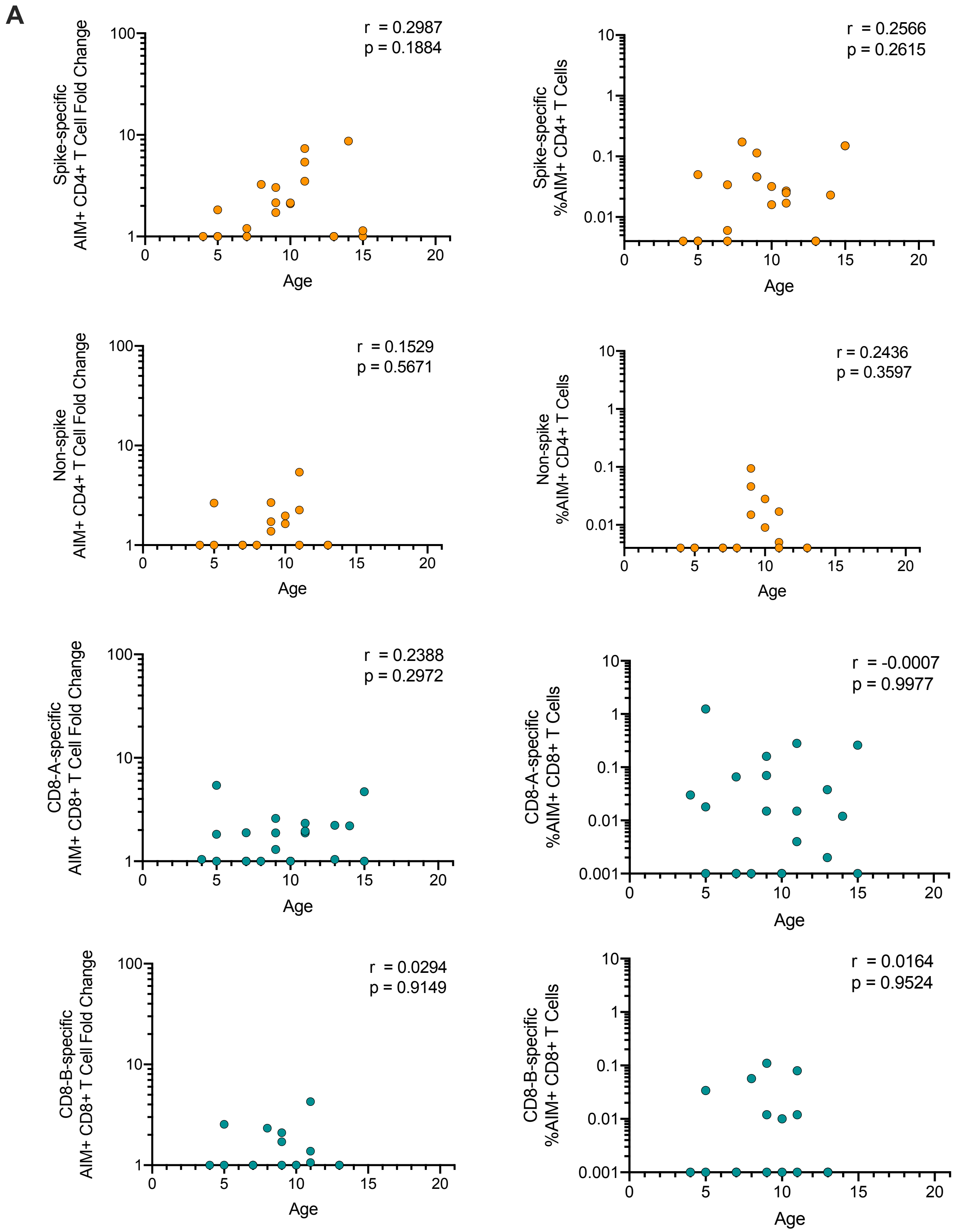
A
r = 0.2987
p = 0.1884
Spike-specific
AIM+ CD4+ T Cell Fold Change
Age
r = 0.2566
p = 0.2615
Spike-specific
%AIM+ CD4+ T Cells
Age
r = 0.1529
p = 0.5671
Non-spike
AIM+ CD4+ T Cell Fold Change
Age
r = 0.2436
p = 0.3597
Non-spike
%AIM+ CD4+ T Cells
Age
r = 0.2388
p = 0.2972
CD8-A-specific
AIM+ CD8+ T Cell Fold Change
Age
r = -0.0007
p = 0.9977
CD8-A-specific
%AIM+ CD8+ T Cells
Age
r = 0.0294
p = 0.9149
CD8-B-specific
AIM+ CD8+ T Cell Fold Change
Age
r = 0.0164
p = 0.9524
CD8-B-specific
%AIM+ CD8+ T Cells
Age

B

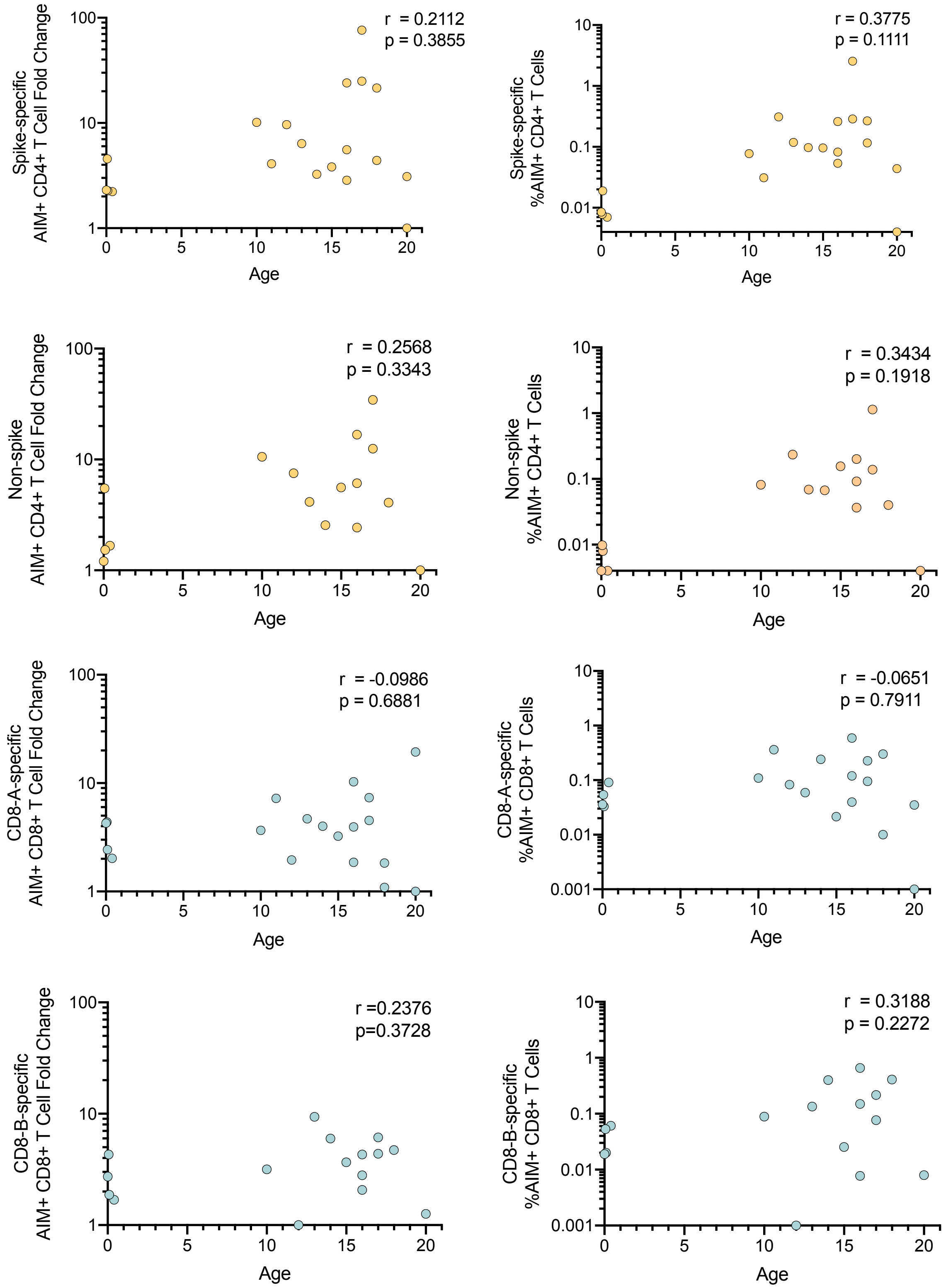

r = 0.2112
p = 0.3855
Spike-specific
AIM+ CD4+ T Cell Fold Change
Age
r = 0.3775
p = 0.1111
Spike-specific
%AIM+ CD4+ T Cells
Age
r = 0.2568
p = 0.3343
Non-spike
AIM+ CD4+ T Cell Fold Change
Age
r = 0.3434
p = 0.1918
Non-spike
%AIM+ CD4+ T Cells
Age
r = -0.0986
p = 0.6881
CD8-A-specific
AIM+ CD8+ T Cell Fold Change
Age
r = -0.0651
p = 0.7911
CD8-A-specific
%AIM+ CD8+ T Cells
Age
r =0.2376
p=0.3728
CD8-B-specific
AIM+ CD8+ T Cell Fold Change
Age
r = 0.3188
p = 0.2272
CD8-B-specific
%AIM+ CD8+ T Cells
Age

Figure S2. Correlation of T-cell Responses by Age. Relationship between SARS-CoV-2-specific CD4+ (Top) and CD8+ (Bottom) T-cell responses by both fold change (L plots) and frequency (R plots) and age for (A) MIS-C and (B) convalescent COVID-19. Statistical relationships were assessed with Spearman's correlation.

Figure S3.

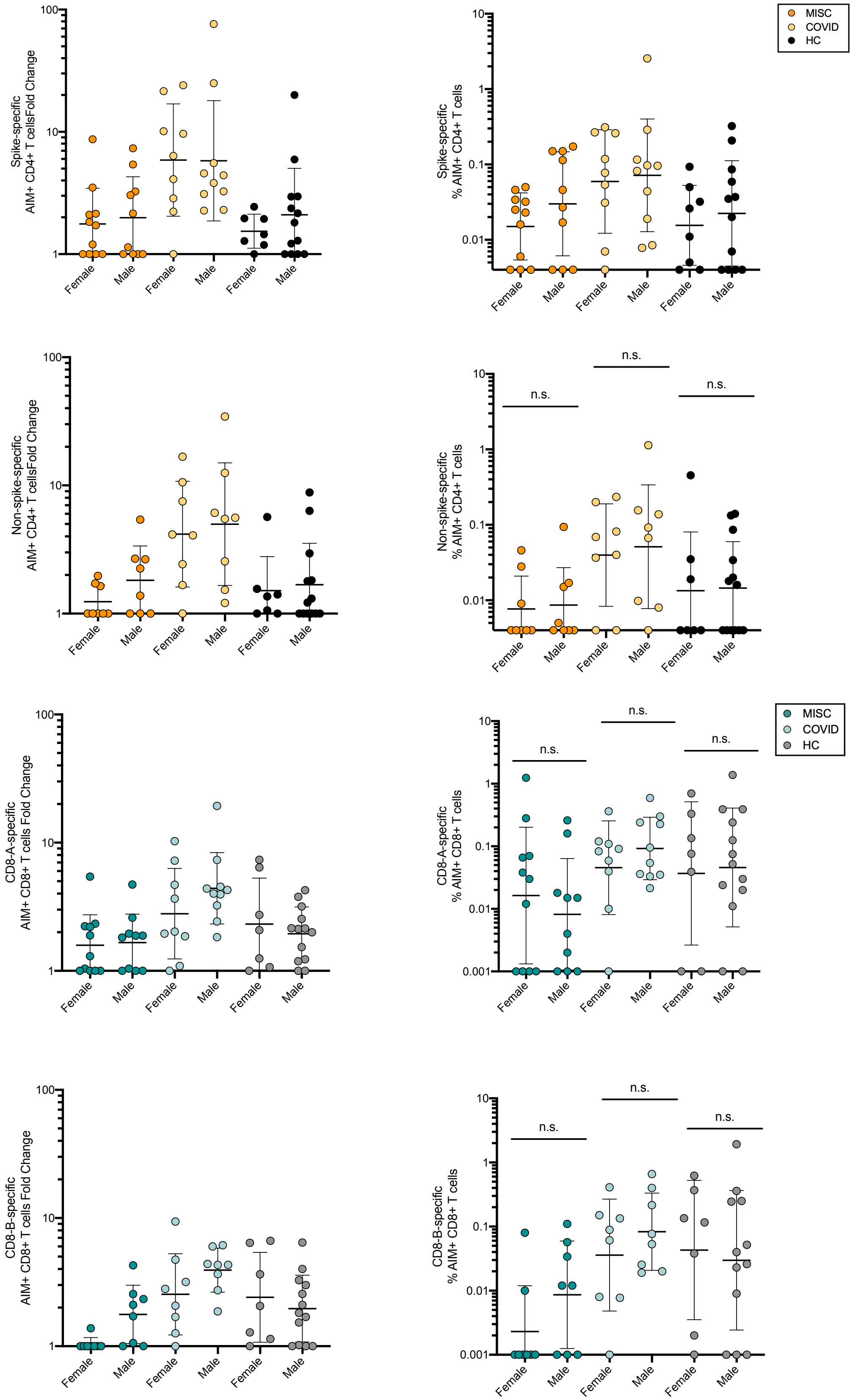

MISC
COVID
HC
Spike-specific
AIM+ CD4+ T cellsFold Change
Spike-specific
% AIM+ CD4+ T cells
Non-spike-specific
AIM+ CD4+ T cellsFold Change
Non-spike-specific
% AIM+ CD4+ T cells
CD8-A-specific
AIM+ CD8+ T cells Fold Change
CD8-A-specific
% AIM+ CD8+ T cells
CD8-B-specific
AIM+ CD8+ T cells Fold Change
CD8-B-specific
% AIM+ CD8+ T cells
n.s.
Female
Male

Figure S3. Distribution of T-cell Responses by Sex. SARS-CoV-2-specific CD4+ (Top) and CD8+ (Bottom) T-cell responses by male and female sex shown as both fold change (L plots) and frequency (R plots). Geometric mean and geometric mean standard deviation (SD) shown with statistical comparison by Kruskal-Wallis test. No significant differences by sex were observed within donor types.

Figure S4.

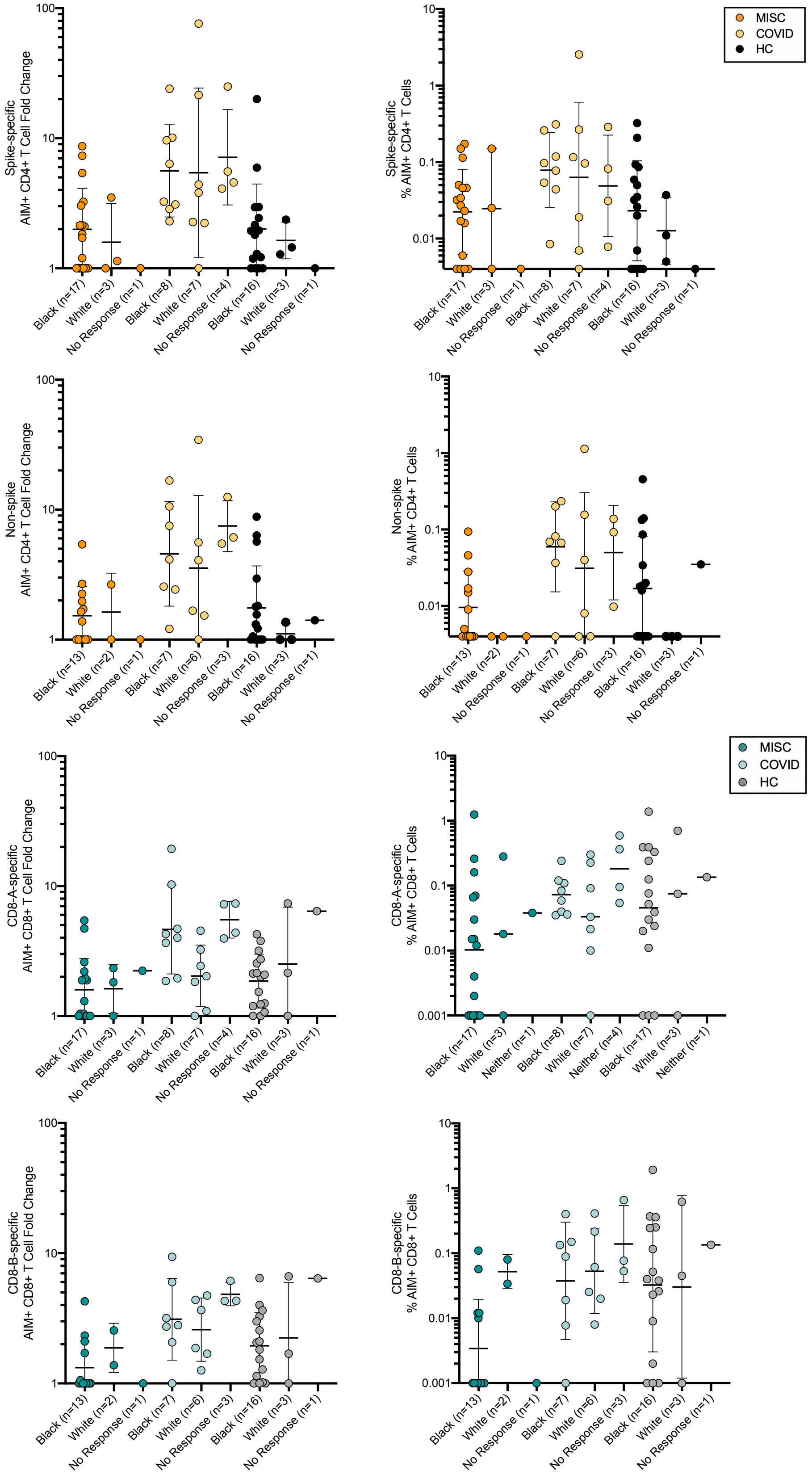
MISC
COVID
HC
Spike-specific AIM+ CD4+ T Cell Fold Change
Spike-specific % AIM+ CD4+ T Cells
Non-spike AIM+ CD4+ T Cell Fold Change
Non-spike % AIM+ CD4+ T Cells
CD8-A-specific AIM+ CD8+ T Cell Fold Change
CD8-A-specific % AIM+ CD8+ T Cells
CD8-B-specific AIM+ CD8+ T Cell Fold Change
CD8-B-specific % AIM+ CD8+ T Cells
Black (n=17)
White (n=3)
No Response (n=1)
Black (n=8)
White (n=7)
No Response (n=4)
Black (n=16)
White (n=3)
No Response (n=1)
Black (n=13)
White (n=2)
Black (n=7)
White (n=6)
No Response (n=3)
Neither (n=1)
Neither (n=4)
Black (n=17)

Figure S4. Distribution of T-cell Responses by Race. SARS-CoV-2-specific CD4+ (Top) and CD8+ (Bottom) T-cell responses by Black, White, or No Response shown as both fold change (L plots) and frequency (R plots). Geometric mean and geometric mean standard deviation (SD) shown with statistical comparison by Kruskal-Wallis test. No significant racial differences were observed within donor type.

Figure S5.

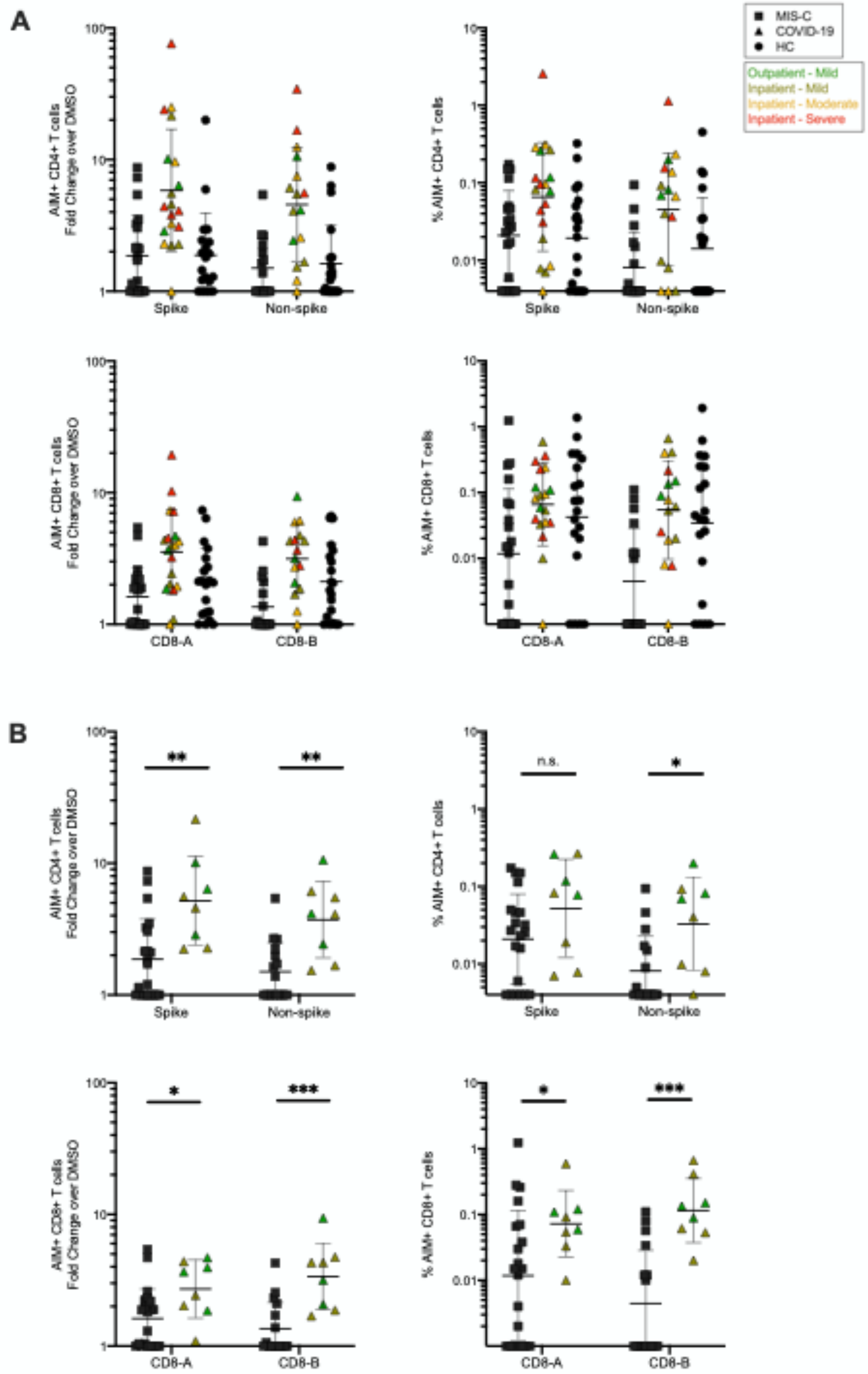
A
B
MIS-C
COVID-19
HC
Outpatient - Mild
Inpatient - Mild
Inpatient - Moderate
Inpatient - Severe
AIM+ CD4+ T cells Fold Change over DMSO
% AIM+ CD4+ T cells
AIM+ CD8+ T cells Fold Change over DMSO
% AIM+ CD8+ T cells
Spike
Non-spike
CD8-A
CD8-B
n.s.
*
**
***

Figure S5. T-cell Responses by COVID-19 Disease Severity. (A) SARS-CoV-2-specific CD4+ and CD8+ T-cell responses (L plots, fold change [FC]; R plots, frequency) by peptide MP and COVID-19 disease severity (categorization defined in Methods). (B) T-cell response data for MIS-C (n=21) and mild COVID-19 (n=8) participants with FC (L plots) and frequency (R plots) shown. Line indicates geometric mean and geometric mean SD. Statistical comparisons conducted with Mann Whitney test. * $p<0.05$, ** $p<0.01$, *** $p<0.001$.

Figure S6.

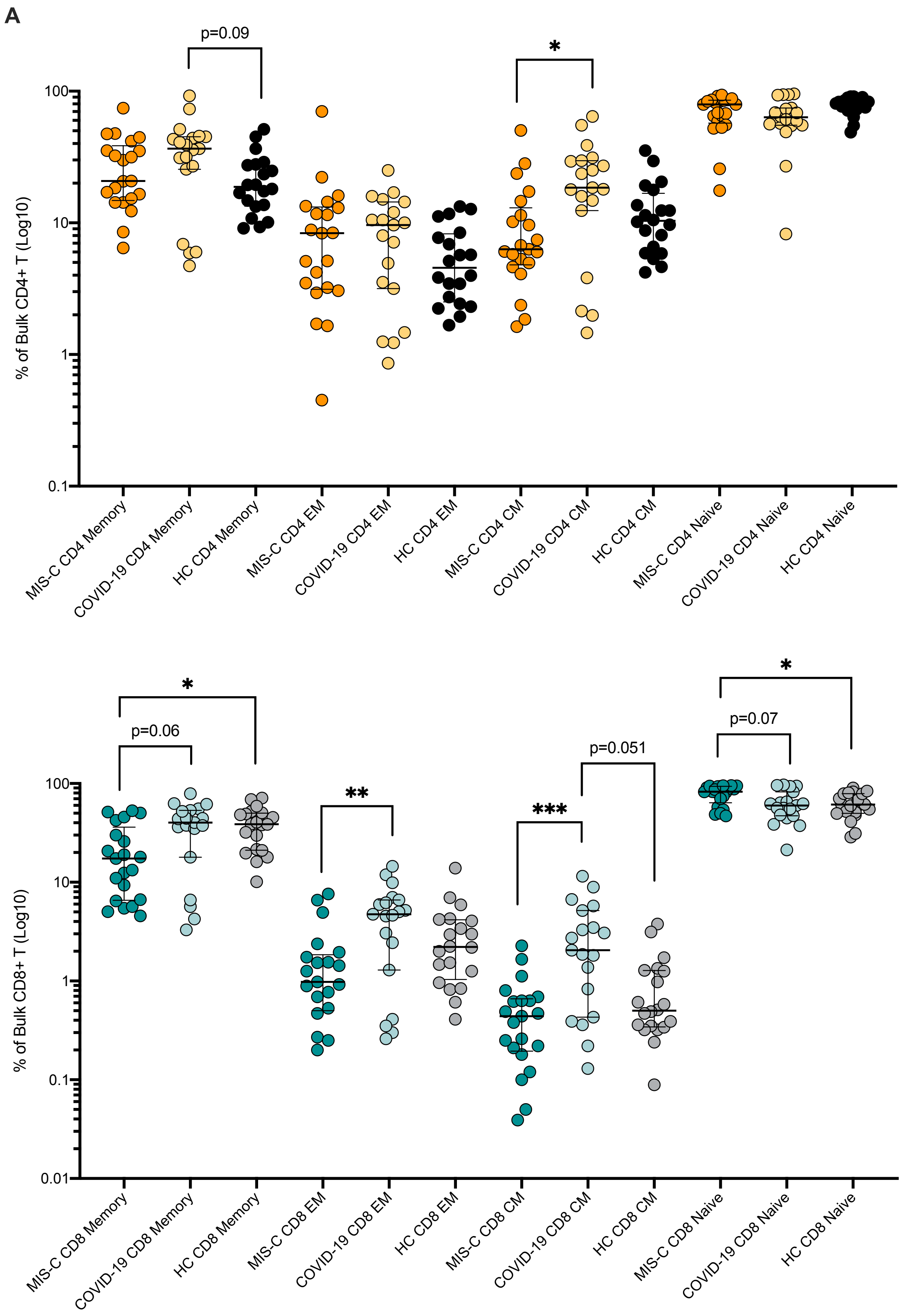
A
p=0.09
*
% of Bulk CD4+ T (Log10)
100
10
1
0.1
MIS-C CD4 Memory
COVID-19 CD4 Memory
HC CD4 Memory
MIS-C CD4 EM
COVID-19 CD4 EM
HC CD4 EM
MIS-C CD4 CM
COVID-19 CD4 CM
HC CD4 CM
MIS-C CD4 Naive
COVID-19 CD4 Naive
HC CD4 Naive
*
p=0.06
**
***
p=0.051
*
p=0.07
% of Bulk CD8+ T (Log10)
100
10
1
0.1
0.01
MIS-C CD8 Memory
COVID-19 CD8 Memory
HC CD8 Memory
MIS-C CD8 EM
COVID-19 CD8 EM
HC CD8 EM
MIS-C CD8 CM
COVID-19 CD8 CM
HC CD8 CM
MIS-C CD8 Naive
COVID-19 CD8 Naive
HC CD8 Naive

B

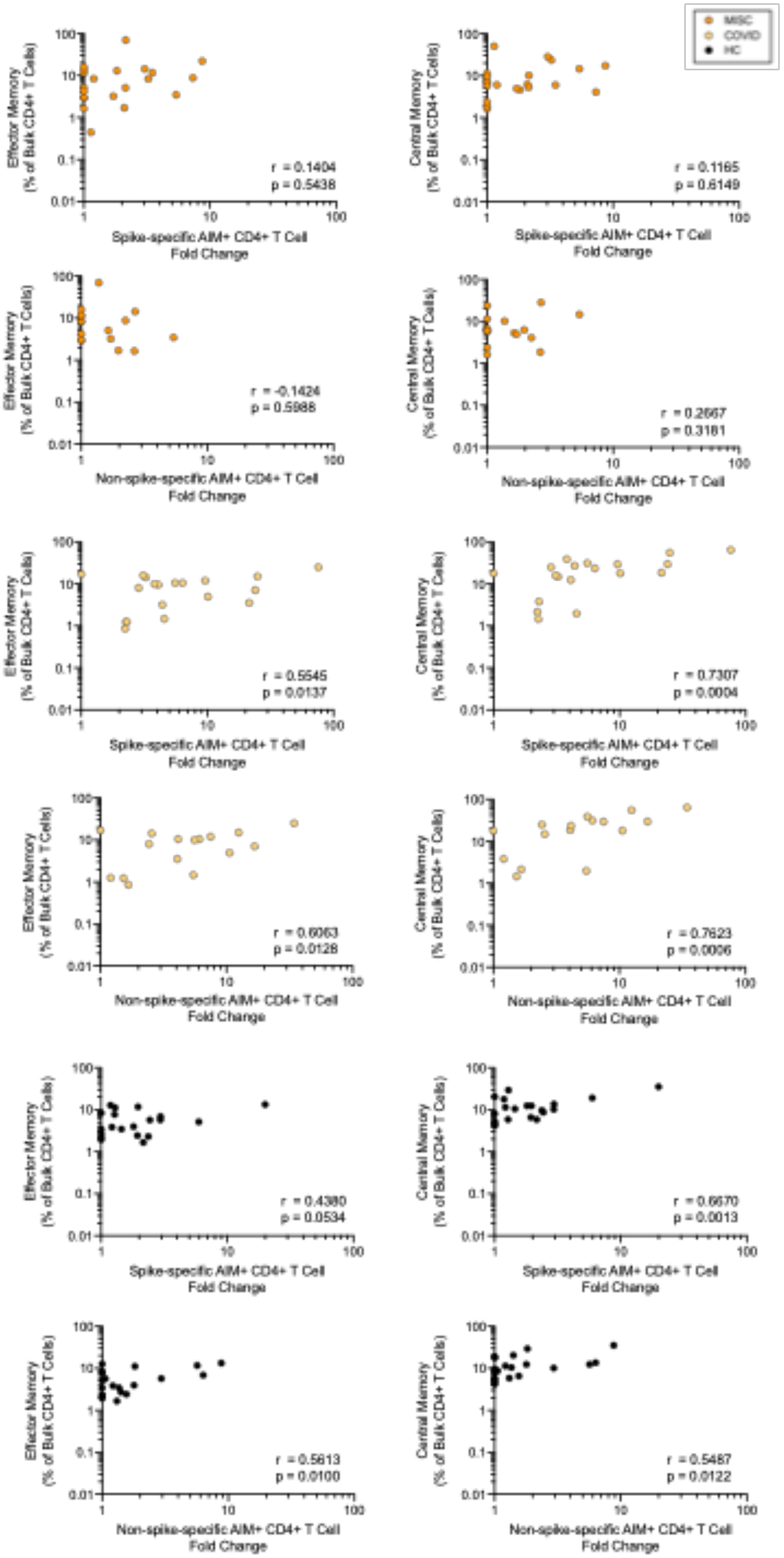
MISC
COVID
HC
Effector Memory (% of Bulk CD4+ T Cells)
Central Memory (% of Bulk CD4+ T Cells)
Spike-specific AIM+ CD4+ T Cell Fold Change
Non-spike-specific AIM+ CD4+ T Cell Fold Change
r = 0.1404
p = 0.5438
r = 0.1165
p = 0.6149
r = -0.1424
p = 0.5988
r = 0.2667
p = 0.3181
r = 0.5545
p = 0.0137
r = 0.7307
p = 0.0004
r = 0.6063
p = 0.0128
r = 0.7623
p = 0.0006
r = 0.4380
p = 0.0534
r = 0.6670
p = 0.0013
r = 0.5613
p = 0.0100
r = 0.5487
p = 0.0122

Figure S6. Distribution of T-cell Subsets by Donor. (A) Bulk CD4+ and CD8+ T-cell memory, effector memory (EM: CCR7-CD45RA-), central memory (CM: CCR7+ CD45RA-), and naïve (CCR7+CD45RA+) T-cells shown as proportions of respective bulk CD4+ and CD8+ T-cells. Median and interquartile range shown with statistical comparison by Kruskal-Wallis test. * $p<0.05$, ** $p<0.01$, *** $p<0.001$. (B) Relationships between SARS-CoV-2-specific CD4+ T-cell responses (fold change) and EM and CM CD4+ T-cell subsets among MIS-C (Top), convalescent COVID-19 (Middle), and HC (Bottom) donors. Statistical relationships were assessed with Spearman's correlation.

Figure S7.

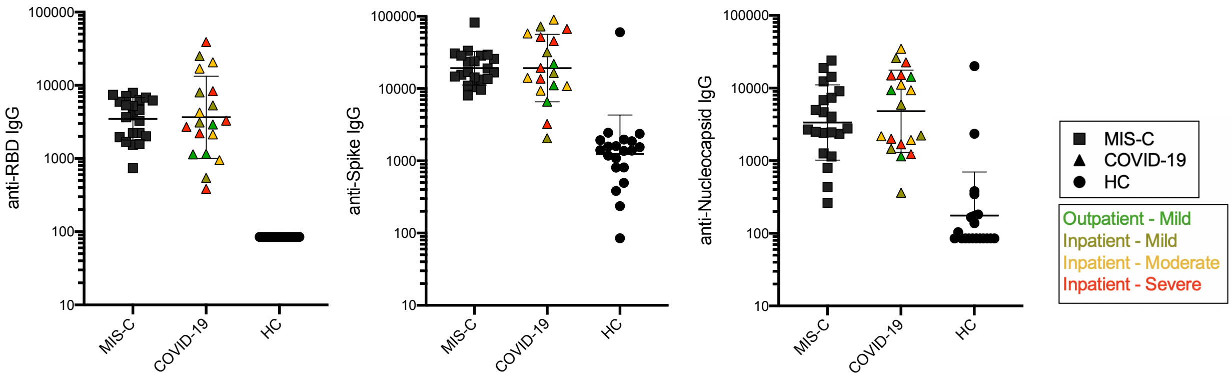

Figure S7. Serologic Responses by COVID-19 Disease Severity. Antibody titers to SARS-CoV-2 RBD, full-length spike, and nucleocapsid protein are shown with COVID-19 disease severity indicated (categorization defined in Methods). The line indicates geometric mean and geometric mean SD.

Figure S8.

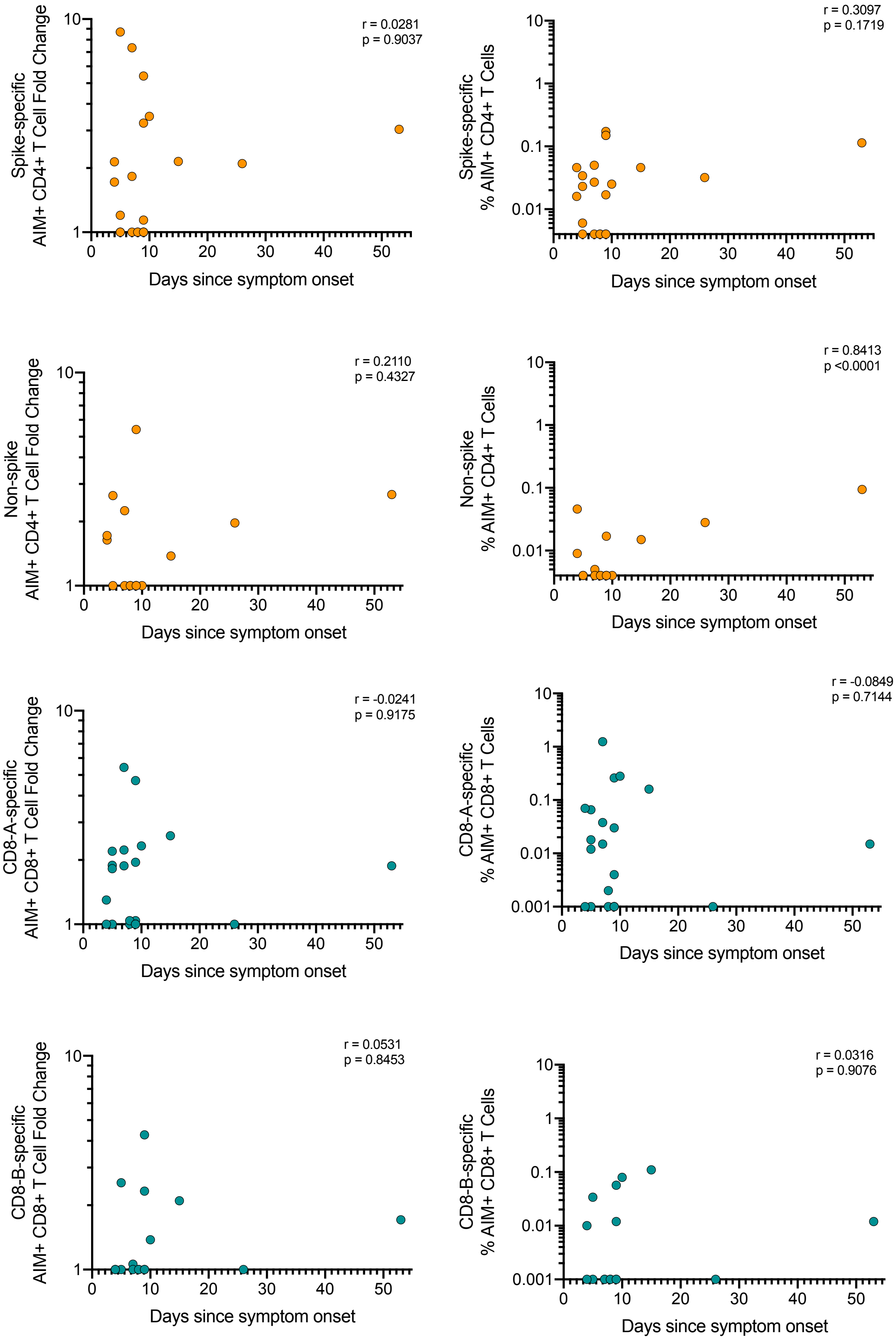

r = 0.0281
p = 0.9037
Spike-specific
AIM+ CD4+ T Cell Fold Change
Days since symptom onset
r = 0.3097
p = 0.1719
Spike-specific
% AIM+ CD4+ T Cells
Days since symptom onset
r = 0.2110
p = 0.4327
Non-spike
AIM+ CD4+ T Cell Fold Change
Days since symptom onset
r = 0.8413
p <0.0001
Non-spike
% AIM+ CD4+ T Cells
Days since symptom onset
r = -0.0241
p = 0.9175
CD8-A-specific
AIM+ CD8+ T Cell Fold Change
Days since symptom onset
r = -0.0849
p = 0.7144
CD8-A-specific
% AIM+ CD8+ T Cells
Days since symptom onset
r = 0.0531
p = 0.8453
CD8-B-specific
AIM+ CD8+ T Cell Fold Change
Days since symptom onset
r = 0.0316
p = 0.9076
CD8-B-specific
% AIM+ CD8+ T Cells
Days since symptom onset

Figure S8. Correlation of T-cell Responses by Days since Symptom Onset in MIS-C Patients. Relationship between SARS-CoV-2-specific CD4+ (Top) and CD8+ (Bottom) T-cell responses by both fold change (L plots) and frequency (R plots) and days since symptoms onset MIS-C (n=21). Statistical relationships were assessed with Spearman's correlation.

Figure S9.

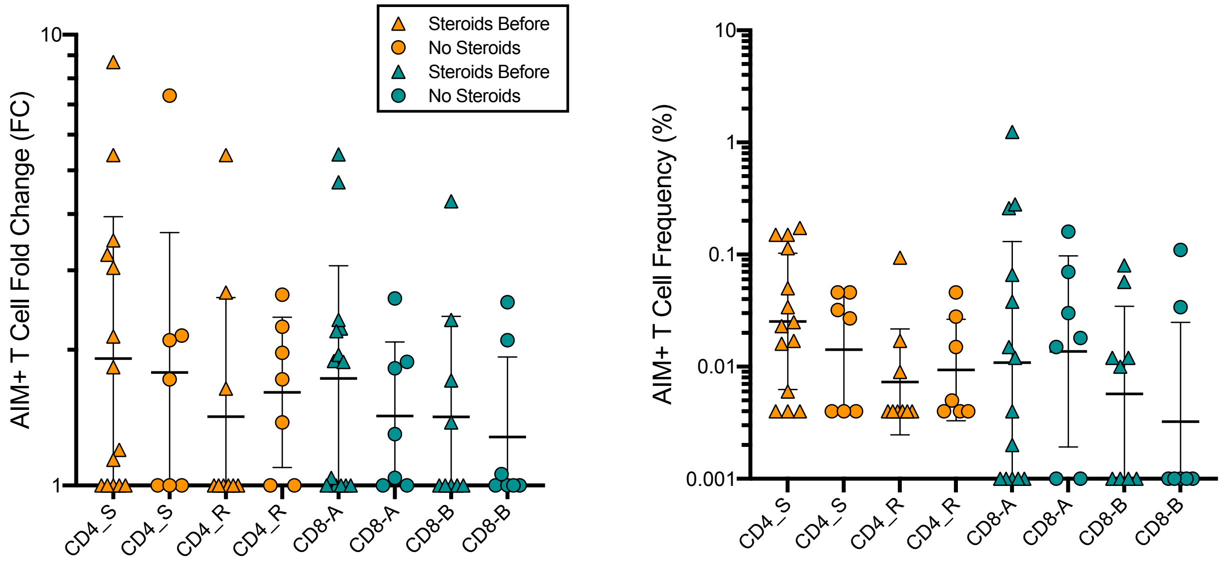

Figure S9. Distribution of T-cell Responses by Steroid Treatment in MIS-C Patients. SARS-CoV-2-specific CD4+ and CD8+ T-cell responses by those who received steroids prior enrollment (n=14) and those who did not (n=7) shown as both fold change (L plots) and frequency (R plots). Geometric mean and geometric mean standard deviation (SD) shown with statistical comparison by Kruskal-Wallis test. No significant differences were observed within MPs by steroid treatment.
